# Advancing fecal volatilome profiling by comprehensive two-dimensional gas chromatography-time-of-flight mass spectrometry and image pattern recognition

**DOI:** 10.1007/s00216-025-06280-6

**Published:** 2026-01-08

**Authors:** Fulvia Trapani, Andrea Caratti, Erica Liberto, Luca Cocolin, Ilaria Goitre, Valentina Ponzo, Simona Bo, Chiara Cordero, Ilario Ferrocino

**Affiliations:** 1 Dipartimento Di Scienza E Tecnologia del Farmaco, Università Di Torino, Via Giuria 9, 10125 Turin, Italy; 2 Dipartimento Di Scienze Agrarie, Forestali E Alimentari, Università Di Torino, Largo P. Braccini 2, 10095 Grugliasco (TO), Italy; 3 Dipartimento Di Scienze Mediche, Università Di Torino, Corso Dogliotti 14, 10125 Turin, Italy

**Keywords:** Comprehensive two-dimensional gas chromatography, Fecal volatilomics, UT fingerprinting, Non-celiac gluten sensitivity, Image pattern recognition

## Abstract

Comprehensive two-dimensional gas chromatography coupled with time-of-flight mass spectrometry (GC×GC-TOFMS) is a powerful technique suited for resolving complex mixtures of volatile organic compounds (VOCs). In this study, GC×GC-TOFMS was combined with an image-based chromatographic fingerprinting strategy to characterize the fecal volatilome of subjects affected by non-celiac gluten/wheat sensitivity (NCGWS) under dietary intervention. Fifty participants underwent a dietary protocol consisting of a gluten-free diet followed by gluten reintroduction while receiving probiotics or placebo. Headspace solid-phase microextraction (HS-SPME) conditions were specifically optimized to maximize the extraction coverage of volatiles, while a combined untargeted–targeted fingerprinting approach (*UT fingerprinting*) enabled the mapping of approximately 1000 volatile features in fecal samples. Chemometric analyses, including principal component analysis (PCA) and partial least squares discriminant analysis (PLS-DA), highlighted informative differences in VOC profiles induced by gluten reintroduction and probiotic administration. Around 270 VOCs were annotated, expanding the existing knowledge on the human fecal volatilome by 20%, predominantly comprising microbial fermentation-derived metabolites such as short-chain fatty acid esters, aldehydes, alcohols, terpenes, and aromatic hydrocarbons. PLS-DA models discriminated probiotic-treated individuals from placebo recipients with 89–90% accuracy, confirming the impact of probiotic supplementation on gut metabolic signatures. The integration of GC×GC-TOFMS and image-based chromatographic fingerprinting effectively captured subtle metabolic variations, demonstrating its suitability in biomarker discovery for dietary interventions and personalized nutrition studies.

**Graphical abstract:**

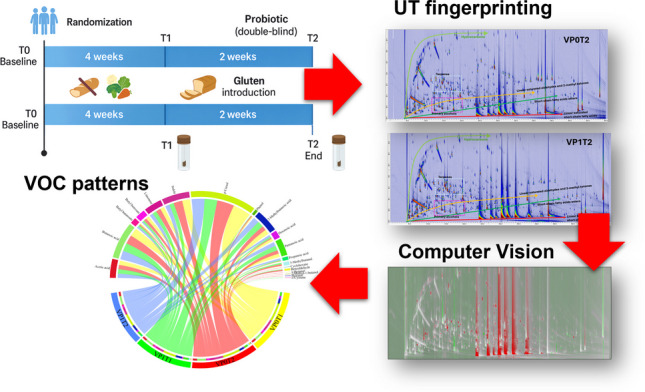

**Supplementary Information:**

The online version contains supplementary material available at 10.1007/s00216-025-06280-6.

## Introduction

Comprehensive two-dimensional gas chromatography coupled to time-of-flight mass spectrometry (GC$\times$GC-TOFMS) has emerged as a cutting-edge analytical technique that provides exceptional resolution, sensitivity, and dynamic range, making it invaluable for the characterization of complex samples with high chemical dimensionality [1]. Unlike traditional one-dimensional (1D) gas chromatography (GC), GC$\times$GC-TOFMS utilizes two distinct columns with different stationary phases, connected in series by a modulator, to achieve enhanced separation of analytes [2, 3]. The comprehensive combination of the two separation dimensions significantly reduces co-elution and generates peak patterns with a rational retention logic based on the sample’s chemical dimensionality [1]. The resulting chromatographic fingerprint [4] takes further advantage of the band compression in space generated by the thermal modulation that enhances peak capacity, further improving resolution and fingerprint information capacity [6]. Moreover, thermal modulation might achieve an enhancement of the signal-to-noise ratio up to ten times 1D-GC in translated conditions allowing for the detection of a wide range of concentrations, from trace levels to highly abundant compounds [5]. This broad dynamic range is particularly valuable in metabolomics, where the ability to detect small changes in complex biological matrices is crucial [6, 7].

In addition to its well-established role in food volatilomics, GC$\times$GC-TOFMS is being increasingly applied to biological fluids such as urine, saliva, serum and breath, further confirming its value in biomedical research [8–10]. These biological matrices, rich in volatile organic compounds (VOCs), serve as essential indicators of health status and disease progression. For example, in urine analysis, GC×GC-TOFMS has been employed to explore the volatilome, uncovering metabolic signatures from dietary intervention, that could also aid in the detection of disease states [11]. GC×GC-TOFMS is also fundamental in analyzing VOCs from saliva and serum, where it provides detailed separation and identification of metabolites crucial for monitoring therapeutic responses or detecting early biomarkers of disease [9, 11, 12]. Breath analysis, which benefits from the technique’s high sensitivity and ability to detect very small concentrations of VOCs, has emerged as a non-invasive diagnostic tool for conditions like lung cancer and metabolic syndromes [13].

Despite the widespread application of GC×GC-TOFMS in metabolomics and nutrimetabolomics, several recent studies have begun to apply this technique to the human fecal volatilome, providing important methodological insights [14–16]. The fecal volatilome, comprising a wide array of VOCs, reflects the metabolic activities of humans and their gut microbiota [11, 16, 17]. Human feces, primarily composed of water, undigested food particles, nitrogen and protein matter, carbohydrates, lipids, and a complex mixture of microbial metabolites, serve as an exceptional matrix for studying the gut microbiome and metabolic diseases, reflecting the interplay between diet, microbiota, and host physiology [8, 18, 19]. In fecal samples, the complex metabolic activity of the gut microbiota drives extensive fermentation processes, generating a wide array of VOCs, many of which are functionally relevant for gut health or serve as potential biomarkers of metabolic dysfunction and disease [20–22]. Among these, short-chain fatty acids (SCFAs) play a crucial role in maintaining intestinal homeostasis, supporting epithelial barrier integrity and exerting anti-inflammatory effects, thereby contributing to overall gut health [23]. In fact, class-specific shifts in the fecal volatilome have been reported across various disorders, indicating that VOC classes may be considered non-invasive markers of underlying pathophysiology. For instance, in a recent study of Alzheimer’s disease, patients were reported to have higher levels of fatty acids and esters in their feces, while terpenes, sulfur compounds, and certain aldehydes were more prevalent in healthy controls [24]. These observations are also consistent with findings from gastrointestinal and metabolic diseases. In ulcerative colitis, significant differences in fecal VOCs were observed compared to healthy subjects [25]. Moreover, recent evidence shows that fecal VOC profiles can even predict late-onset sepsis, reflecting gut microbial status and disease progression [26, 27]. Overall, several studies support the suitability of GC×GC-TOFMS for fecal volatilomics and highlight the potential of fecal VOCs to serve as discriminant markers across a range of intestinal, metabolic, and neurological conditions.

This study employs GC×GC-TOFMS to conduct a comprehensive profiling of the fecal volatile metabolites using as a test bench the samples from individuals with non-celiac gluten/wheat sensitivity (NCGWS) [28] subjected to a dietary intervention and probiotic implementation. To our knowledge, this analytical platform has never been applied to characterize metabolic responses associated with NCGWS, as well as for the chromatographic fingerprinting with image-based pattern recognition to ensure the in-depth yet comprehensive analysis of the human fecal volatilome in this specific condition. Conditions such as NCGWS are associated with adverse reactions to gluten. Unlike celiac disease, however, these conditions lack clear and validated diagnostic biomarkers, making their study and diagnosis particularly challenging [29]. Metabolomic approaches, especially those involving the characterization of VOCs from fecal samples, offer potential pathways to uncovering metabolic signatures that can be used as biomarkers for these gluten-related disorders [8].

## Materials and methods

### Study design

**Fig. 1:**
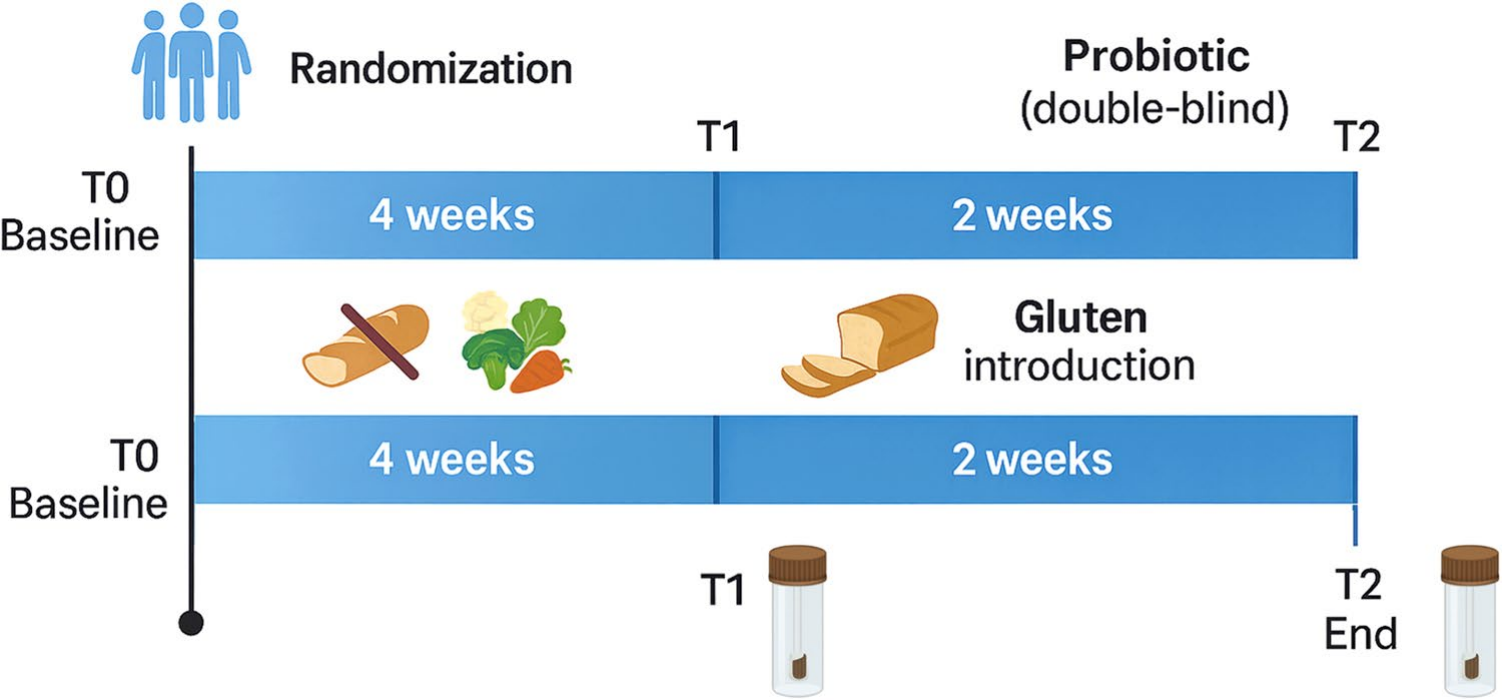
Schematic overview of the study design. After baseline assessment (T0), participants were randomized to receive either probiotic or placebo in a double-blind manner for a total of 6 weeks. The first 4 weeks consisted of a gluten-free diet (T0-T1), followed by fecal sample collection at T1. During the following 2 weeks, gluten was reintroduced into the diet (T1–T2), and fecal samples were collected again at T2 (end of the intervention)

This study consisted of 50 adults reporting symptoms suggestive of NCGSWS as previously described [28]. All participants underwent a double-blind, placebo-controlled crossover gluten challenge to confirm gluten sensitivity. The blind challenge identified 27 participants as gluten-responsive, who were then randomized into two arms: 13 to the probiotic group and 14 to the placebo group. The experimental arm consisted of a 6-week daily intake of commercial probiotics (*Lactiplantibacillus plantarum* P17630 4.9 × 109, *Lacticaseibacillus paracasei* I1688 1 × 109, *Ligilactobacillus salivarius* I1794 2.5 × 106; Progefarm, SrL, Novara®), while the control arm received a placebo for the same duration. All participants followed a gluten-free diet for four weeks (sampling point T1), after which gluten was reintroduced into their diets for two weeks (sampling point T2). Shotgun metagenomic data are outside the scope of this manuscript and are presented in a previous study [28]. Samples were coded prior to analysis to preserve blinding. Each record includes a sample code, treatment arm (VP0 = placebo; VP1 = probiotics), and time point (T1, T2). For interpretability, we also report a combined factor (VP0T1, VP1T1, VP0T2, VP1T2) and the diet phase associated with each time point (gluten-free diet up to T1; free diet between T1 and T2). The total number of fecal samples available for volatilomics was 54; they were analyzed in triplicate following a random order. Figure 1 shows a schematic overview of the study design, while the full sample mapping is provided in the Supplementary Table – ST1. In addition, food frequency and general health-related quality of life questionnaires were assessed. The study was conducted at AOU Citta della Salute e della Scienza di Torino (University of Turin, Turin, Italy) in accordance with the Declaration of Helsinki, and all procedures were approved by an appropriate ethics committee. Full details of the study design and procedures can be found in the primary study reference [28] and at www.clinicaltrials.gov (identifier: NCT06884241).

### Fecal sample collection and handling

Fecal samples from study participants were self-collected in sterile polypropylene containers, maintained at 4 °C during transport, and processed within 24 h of collection. The procedure was validated by Ponzo et al*.* in a previous study and demonstrated to be suitable for preserving samples’ integrity for nucleic acid extraction and volatilomics [30]. Specimens of about 10 g were homogenized under aseptic conditions in a cryo-mortar in the presence of liquid nitrogen to avoid volatile analytes’ losses and then aliquoted and stored at −80 °C until analysis. A quality control matrix was prepared from a pooled mice fecal homogenate, processed, and stored identically to study specimens. Samples were provided by the Pharmacology Unit of the Department of Drug Science and Technology (University of Turin, Turin, Italy), using fecal material from healthy control mice enrolled in ethically approved studies. The quality control sample was analyzed at the beginning of each analytical sequence and at regular intervals throughout the batch to verify system suitability, monitor instrumental stability, retention reproducibility, and support response normalization through internal-standard referencing. The high analytical repeatability was supported by the tight clustering of quality control (QC) samples in the PCA space (Supplementary Fig. 1 – SF1), highlighting minimal instrumental variability across runs. On average, approximately 1000 distinct chromatographic features were detected in the QC sample, confirming the high chemical complexity of the fecal volatilome. Among these, around 270 VOCs were annotated based on retention (i.e., linear retention index *I*^*T*^ [31], tolerance ± 20 units) and spectral matching (i.e., NIST similarity algorithm, direct match factor (DMF) > 950) and were further considered as *targeted* variables throughout the study [32]. Where available, reference standards were used for identity confirmation.

### Reference compounds and chemicals

Pure standards of *n-*alkanes (from n-C9 to n-C25) for system evaluation and *I*^*T*^ calibration, internal standards (ISs) α-/β-thujone and methyl 2-octynoate for response normalization, and pure reference compounds for targeted analytes’ identity confirmation were supplied by Merck (Milan, Italy). Cyclohexane (HPLC grade) for *n-*alkanes standard solution (at 100 mg/L) and pure dibutyl phthalate for ISs working solutions (at 100 mg/L) were also from Merck (Milan, Italy).

### VOC sampling and analysis by HS‐SPME–GC×GC-TOFMS

Headspace solid-phase microextraction (HS-SPME) was used for fecal VOC sampling with an SPR autosampler for GC (SepSolve-Analytical, Llantrisant, UK). A divinylbenzene/carboxen/polydimethylsiloxane (DVB/CAR/PDMS) fiber (d_f_ 50/30 µm; 2 cm length; Merck, Bellefonte, PA, USA) was selected based on the fiber-screening results (”) and conditioned according to the manufacturer’s instructions. Before VOC extraction, 5 µL of ISs α/β-thujone and methyl 2-octynoate (100 mg L⁻^1^ in dibutyl phthalate) were spiked in the sampling vial (20 mL) containing fecal material. For each extraction, 0.200 ± 0.005 g of homogenized fecal material was precisely weighed in a 20-mL headspace vial sealed and equilibrated at 40 °C under constant agitation (500 rpm) for 50 min. The extraction temperature of 40 °C was selected as it closely approximates physiological conditions and minimizes thermal degradation or formation of VOCs that can occur at higher temperatures, thus preserving the native volatilome profile. The extraction time of 50 min represented the maximum feasible duration compatible with the subsequent chromatographic run, providing adequate adsorption/partition of analytes on the fiber without compromising sample integrity. Immediately after extraction, the fiber was transferred to the split/splitless injector port maintained at 270 °C, and thermal desorption was performed for 5 min in split mode (1:20). All extractions were performed in triplicate and randomized over 2 weeks to assess repeatability.

Method precision was evaluated by the percentage relative standard deviation (%RSD) on retention times and signal-to-noise ratio (SNR) of selected 2D peaks. Daily quality controls (QC; *n* = 4) monitored system stability, with QC vials prepared and analyzed under identical conditions. It is important to note that the use of IS in this context serves solely for signal normalization and does not enable absolute quantification. This limitation arises from the inherent variability in analyte–fiber partitioning and matrix effects associated with HS-SPME sampling.

### GC×GC-TOFMS instrument configuration with thermal modulation

Analyses employed an Agilent 7890B GC coupled to a BenchTOF‐Select TOFMS (Markes International, UK). The transfer line and ion source were maintained at 270 °C. Electron ionization was performed at 70 eV; mass range was 40–350 *m/z* at 100 Hz. The system was equipped with a two-stage KT 2004 loop type thermal modulator (Zoex Corporation, Houston, TX) cooled with liquid nitrogen and controlled by Optimode v2.0 (SRA Instruments, Cernusco sul Naviglio, Milan, Italy). Modulation period (*P*_*M*_) was set at 4 s, with a hot jet pulse time of 250 ms. A mass flow controller (MFC) reduced the cold-jet stream from 40 to 5% of the total flow with a linear function along the run duration.

The column setup comprised a ^1^D HeavyWax™ column (30 m $\times$ 0.25 mm i.d. $\times$ 0.25 µm *d*_*f*_, PEG) from J&W (Agilent, Little Falls, DE, USA) and a ^2^D OV1701 column (1.0 m $\times$ 0.10 mm i.d. $\times$ 0.10 µm *d*_*f*_, (86% polydimethylsiloxane, 7% phenyl, 7% cyanopropyl) from Mega (Legnano, Milan, Italy). Connections used a 1.0 m $\times$ 0.1 mm fused-silica capillary loop and SilTite μ-unions. The injector operated at 250 °C in split mode (1:20) with a dedicated SPME liner. Helium carrier flowed at 1.3 mL min⁻^1^; oven ramped from 40 (1 min) to 260 °C at 3.5°C min⁻^1^. No secondary oven was adopted in the system setup. The *n-*alkanes liquid sample solution for *IT* determination was analyzed under the following conditions: split/splitless injector in split mode, split ratio of 1:20, injector temperature of 250 °C, and injection volume of 1 µL.

### The UT fingerprinting approach: principles and analytical workflow

The combined untargeted and targeted (*UT*) *fingerprinting* strategy implemented in this study uses the comprehensive capabilities of GC×GC-TOFMS to generate detailed and reproducible VOC profiles from complex biological matrices such as feces. This approach enables both the exhaustive exploration of the detectable volatilome (untargeted components) and selective detection of known metabolites of interest (targeted components), improving the interpretability and diagnostic potential of chromatographic data. The core principle relies on the automated generation of a feature map (i.e., template) composed of reliable 2D peaks and broader peak-region objects, which are used to extract consistent chemical information across the full set of samples [33–35]. These templates are generated by an automated workflow in GC Image Investigator™ (GC Image) that applies a series of computational steps to extract reproducible VOC patterns from a dataset of matched chromatograms [36].

The process begins with the selection of a subset of representative chromatograms capturing the variability of the VOC patterns. These are subjected to automated 2D peak detection, using a SNR threshold of 50 and spectral similarity constraints (DMF and RMF ≥ 700, based on the NIST similarity algorithm [37]) to ensure confident peak matching. Peaks occurring in at least 50% of the selected chromatograms are retained as *reliable features*, following a relaxed but robust criterion previously optimized for food and biological matrices [7, 35]. These features are then used to temporally align and register chromatograms, correcting for shifts in retention time along both dimensions. Registered chromatograms are fused to generate a composite chromatographic image, *cumulative Image*, from which the UT template is built. The template includes individual peaks and delineated peak regions: graphical objects that define the VOC signal footprint and accommodate minor variability in retention and shape. This untargeted feature map enables the reproducible extraction of semi-quantitative data, even in the presence of partially co-eluting compounds. Targeted components are then integrated into the same feature template. These are selected based on strict identification criteria, including spectral similarity thresholds (DMF and RMF ≥ 950) and agreement with experimental *I*^*T*^ (± 20 units vs. tabulated values), following approaches validated in prior studies on complex fractions [34, 35].

The final feature template, consisting of about 1000 detected components, was applied to the full chromatographic dataset for robust tracking, response extraction, and cross-sample alignment of VOC signatures. The resulting feature matrix was used as the basis for multivariate statistical modeling, providing a chemically informative fingerprint of the fecal volatilome under dietary and probiotic intervention.

### Fiber selection and VOCs profiles

HS-SPME was employed to evaluate the extraction performance of seven commercial SPME fibers for fecal volatilome profiling. The choice of fiber coating plays a crucial role in ensuring comprehensive VOC coverage; however, the literature still lacks systematic investigations on the efficiency of different SPME coatings in fecal volatilomics. Although multicomponent fibers are generally expected to be the most efficient, a comprehensive evaluation under standardized extraction conditions (time and temperature) would support future targeted applications focused on volatile markers of interest.

Each fiber was tested in triplicate on pooled fecal QC samples under the standardized conditions described in ” A total of 5.0 µL of an IS solution (α-/β-thujone and methyl 2-octynoate, 100 mg L⁻^1^ in dibutyl phthalate) was spiked into a 20-mL headspace vial to enable normalization of peak volumes and quality control of analytical response. The fibers compared included coatings with different adsorption/absorption properties and film thicknesses: divinylbenzene/carboxen/polydimethylsiloxane (DVB/CAR/PDMS; 50/30 µm, 2 cm), DVB/CAR/PDMS (50/30 µm, 1 cm), divinylbenzene/polydimethylsiloxane (DVB/PDMS; 65 µm, 1 cm), carboxen/polydimethylsiloxane (CAR/PDMS; 50/30 µm, 1 cm), polydimethylsiloxane/divinylbenzene (PDMS/DVB; 65 µm, 1 cm), polydimethylsiloxane (PDMS; 100 µm, 1 cm), and polyacrylate (PA; 85 µm, 1 cm). Results were evaluated based on the number of detected features exceeding a SNR threshold, the SNR distribution of major VOCs, and the chemical class coverage. SNR was calculated as peak height divided by local baseline noise within the corresponding modulation slice; peaks attributable to fiber bleed or other non-sample interferences were removed by blank subtraction and library/retention-logic screening. The use of SNR instead of absolute peak values (e.g., peak height in 1D-GC or 1D-LC) was chosen to provide a more representative measure of the overall sensitivity of the sampling approach. As 2D peaks were considered only when exceeding a SNR threshold of 50, the combination of the number of detected peaks and their cumulative SNR offers a meaningful indication of the method’s coverage and dynamic range.

**Fig. 2:**
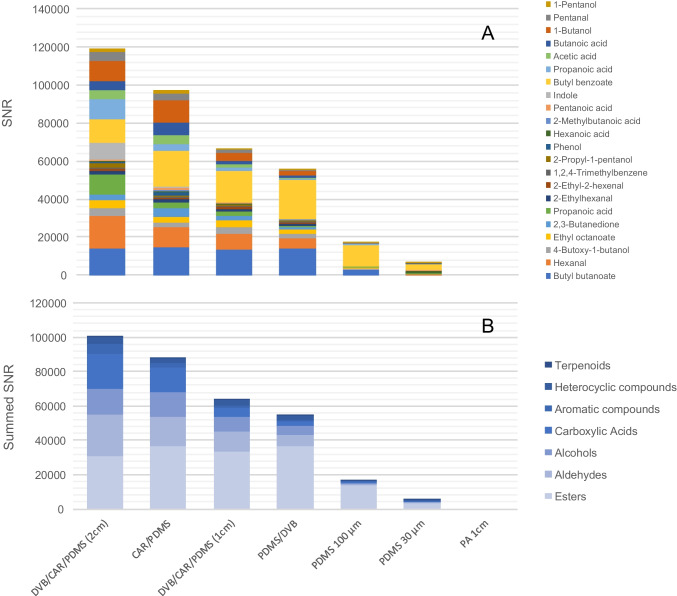
**A** SNR distribution of the twenty highest-response VOCs extracted from fecal headspace using seven different HS-SPME fibers, highlighting compound-specific performance. **B** Summed SNR values by chemical class (e.g., esters, aldehydes, alcohols, carboxylic acids, aromatic and heterocyclic compounds, terpenoids), showing the cumulative extraction efficiency of each fiber across major VOC categories

The DVB/CAR/PDMS fiber (2 cm), as expected, exhibited the best extraction performance (305 features), followed by CAR/PDMS (265 features), DVB/CAR/PDMS (1 cm, 261 features), and PDMS/DVB (250 features). The total number of VOC features detected with SNR over 50 for each fiber is reported in the Supplementary Fig. 2 – SF2. Fibers coated only with PDMS, particularly those with high film thickness (e.g., 100 µm), showed a substantial drop in efficiency (PDMS 100 µm, 60 features; PDMS 30 µm, 89 features), most likely due to the applied constraints in sampling conditions (time and temperature) that do not allow efficient equilibration/sorption of semi-volatiles. Notably, the PA fiber failed to extract any detectable feature with a SNR above 50, suggesting poor interaction with the dominant VOCs in fecal headspace under the given conditions. These data confirm that mixed-phase coatings with both adsorption and absorption capacity (e.g., DVB/CAR/PDMS) are preferable for broad-spectrum VOC capture in complex matrices such as feces. To further assess coating performance and selectivity, based on SNR, the 20 highest-response features per fiber were selected as class-representative VOCs, and their distributions were compared (Fig. 2A). The resulting profiles indicate that DVB/CAR/PDMS (2 cm) provided the largest cumulative SNR and the widest chemical coverage across volatility and functionality domains. DVB/CAR/PDMS (1 cm) and DVB/PDMS (1 cm) exhibited a selective enhancement for esters, consistent with the preferential uptake of mid-volatility, low-polarity analytes, whereas PDMS coatings and PA yielded uniformly lower cumulative SNR and limited class representation. It is also evident that the DVB/CAR/PDMS (2 cm) fiber combines high sensitivity with broad coverage, enabling detection of high-abundance volatiles relevant to fecal volatilome characterization.

Figure 2B reports the class-resolved summed SNR for the twenty highest-SNR VOC features recovered with each HS-SPME fiber. Aggregating SNR by class (esters, aldehydes, alcohols, carboxylic acids, ketones, aromatic compounds, heterocyclic compounds, terpenoids) provides a compact view of coating selectivity and chemical affinity. The DVB/CAR/PDMS (2 cm) fiber achieved the most balanced performance, showing high cumulative responses across all chemical classes. The CAR/PDMS, DVB/CAR/PDMS (1 cm), and PDMS/DVB fibers displayed selective enhancement in ester capture, indicating a higher affinity for medium-volatility, low-polarity compounds in that group. Meanwhile, the PA fiber, again, failed to recover relevant quantities of any chemical class. These findings suggest that while DVB/CAR/PDMS (2 cm) ensures optimal comprehensive coverage for untargeted profiling, other fibers like PDMS/DVB or DVB/CAR/PDMS (1 cm) may be useful for targeted approaches focusing on ester-rich odor profiles. Overall, the comparative data confirm that the DVB/CAR/PDMS fiber (2 cm) is the most effective for global profiling of fecal VOCs. It consistently outperformed all other coatings in terms of the number of features, signal intensity, and chemical class representation. Although other fibers showed useful selectivity for certain compound classes (esters in particular), none matched the overall robustness and versatility of DVB/CAR/PDMS (2 cm) for untargeted volatilome studies. Therefore, this fiber was selected for all subsequent sample analyses.

### Data acquisition, 2D data processing, and statistical analysis software

Raw chromatographic data were acquired using TOF-DS software (Markes International, Llantrisant, UK), integrated with the GC×GC-TOFMS platform. The acquired files were subsequently processed using GC Image GC×GC Software, Version 2025 (GC Image, LLC, Lincoln, NE, USA). Two-dimensional peak detection, feature alignment, and template-based feature tracking were performed within the GC Image Investigator™ environment, following the UT fingerprinting workflow described above. Statistical analysis and multivariate modeling of the extracted feature matrices were carried out using XLSTAT software (Addinsoft, New York, USA), with additional data handling performed in Microsoft Excel 365 (Microsoft Corporation, Redmond, WA, USA). The chord diagram was generated using the “circlize” function of R studio (R Core Team - 2020).

Analytes’ relative amount descriptors adopted for profiling and fingerprinting were first normalized over the ISTDs and then converted to % over the total normalized response from all untargeted and targeted (UT) peak features.

For multivariate modelling, the normalized data matrix was mean-centered before analysis. Partial least squares-discriminant analysis (PLS-DA) was performed using a Monte Carlo cross-validation scheme based on repeated stratified random hold-out partitions, in which approximately 90% of the samples were used for model calibration, and the remaining 10% were reserved as an external validation subset at each iteration. The validation subset preserved the class distribution of the whole dataset and typically comprised ten samples. This procedure was repeated 100 times to ensure robustness and to minimize bias related to sample partitioning. Feature selection was based on the Fisher ratio (*F* > 6, *α* = 0.05), retaining only variables showing significant between-class variance. The mean cross-validated classification accuracy was approximately 89%, confirming the stability and predictive reliability of the model across iterations.

## Results and discussion

### VOCs composition: untargeted and targeted fingerprinting results and chemical classification

Comprehensive two-dimensional GC-TOFMS allowed us to capture the extensive complexity of the fecal volatilome [1]. Untargeted fingerprinting revealed approximately 1000 volatile features consistently present in all samples (i.e., reliable peaks). Among these, about 270 compounds could be consistently annotated; they are listed in Table 1 together with CAS registry numbers, retention times (^1^*t*_*R*_ and ^2^*t*_*R*_), relative standard deviation on retention times calculated over all analytical runs (RSD%), *I*^*T*^ experimentally estimated, and *I*^*T*^ tabulated in the NIST spectral library and/or in the NIST Chemistry WebBook. The listed information also indicates whether each compound has previously been reported in the literature as a characteristic VOC in fecal samples [11].

**Table 1:** List of targeted compounds annotated based on *I*^*T*^ and spectral similarity together with averaged retention times (^*1*^*t*_*R*_ and ^*2*^*t*_*R*_), relative standard deviation (RSD%) calculated aver all samples, *I*^*T*^ experimentally estimated and *I*^*T*^ tabulated in NIST spectral library. For features whose fragmentation pattern matched the corresponding spectral library entry with a DMF above 950, but whose *I*^*T*^ did not fall within the tolerance window, only the compound class is reported followed by a numerical code. Analytes reported in the comprehensive review of human VOCs [11] are annotated with a (✓)

Compound name	CAS	^*1*^ *t*_*R*_(min)	RSD%	^*2*^ *t*_*R*_(sec)	RSD%	*I* ^*T*^ exp	*I* ^*T*^ tab	Reported in [11]
Trimethylamine	75-50-3	4.13	0.31	0.25	6	622	609	✓
Heptane	142-82-5	4.53	0.41	0.55	3.32	700	700	
Cyclohexane	110-82-7	4.67	0.27	0.49	2.7	722	732	
Dimethyl sulfide	75-18-3	4.73	0.21	0.32	2.6	789	773	✓
Acetaldehyde	75-07-0	4.83	0.43	0.24	2.18	799	780	✓
Octane	111-65-9	5.07	0.14	0.9	2.62	800	800	
Propanal	123-38-6	5.08	0.42	0.32	2.07	814	800	✓
Acetone	67-64-1	5.20	0.31	0.33	1.64	822	814	✓
2-Methylpropanal	78-84-2	5.20	0.74	0.39	3.99	834	830	✓
2-Methylfuran	534-22-5	5.80	0.25	0.39	1.63	866	871	✓
Butanal	123-72-8	5.87	0.24	0.44	2.12	870	877	✓
3-Methylfuran	930-27-8	6.07	0.2	0.41	2.49	881	853	✓
2-Propanol	67-63-0	6.12	0.41	0.31	4.23	898	915	✓
2-Butanone	78-93-3	6.13	0.5	0.46	4.18	904	905	✓
Ethanol	64-17-5	6.53	0.22	0.28	1.31	906	920	✓
3-Methyl-2-butanone	563-80-4	6.56	0.12	0.58	1.9	909	929	✓
Methyl trimethylacetate	598-98-1	6.65	0.32	0.66	5.31	911	887	
3-Methylbutanal	590-86-3	6.40	0.07	0.56	5.92	918	916	✓
2,3-Butanedione	431-03-8	7.47	0.36	0.4	10.53	955	978	✓
Ethyl propanoate	105-37-3	7.58	0.65	0.61	6	957	966	✓
Pentanal	110-62-3	7.64	0.52	0.62	1.88	972	984	✓
1-Vinylaziridine	921-91-1	7.80	0.11	0.56	1.18	973	ND	
Allyl methyl sulfide	10152-76-8	8.07	0.13	0.63	2.66	981	970	✓
Ethyl isobutanoate	97-62-1	8.09	0.52	0.73	4.61	982	971	✓
4-Methyl-2-pentanone	108-10-1	8.11	0.41	0.76	3.81	984	1010	✓
Acetonitrile	75-05-8	8.12	0.16	0.31	2.75	984	1012	✓
Methyl butanoate	623-42-7	8.18	0.6	0.63	4.34	989	989	✓
3-Methyl-2-pentanone	565-61-7	8.20	0.28	0.8	3.9	995	1016	✓
Decane	124-18-5	8.28	0.03	2.26	6.94	1000	1000	✓
α-Pinene	80-56-8	8.33	0.26	1.53	6.25	1002	1015	✓
α-Thujene	02/02/2877	8.40	0.17	1.44	6.8	1005	1015	✓
1-Propanol	71-23-8	8.53	0.33	0.34	1.99	1012	1030	✓
2-Butanol	78-92-2	8.55	0.32	0.39	3.58	1020	1019	✓
Methyl isovalerate	556-24-1	8.63	0.4	0.78	4.55	1022	1022	✓
3-Hexanone	589-38-8	9.00	0.2	0.86	4.85	1037	1040	✓
Ethyl butanoate	105-54-4	9.00	0.75	0.86	4.13	1037	1044	✓
(E)-2-Butenal	4170-30-3	9.03	0.25	0.5	2.75	1042	1046	✓
2,3-Pentanedione	600-14-6	9.20	0.27	0.55	2.29	1048	1056	✓
Ethyl 2-methylbutanoate	7452-79-1	9.24	0.43	1.08	3.41	1054	1062	✓
Isopropenyl ethyl ketone	565-69-5	9.40	0.44	0.74	4.79	1059	1069	✓
Dimethyl disulfide	624-92-0	9.53	0.44	0.63	4.12	1066	1065	✓
5-Methyl-3-hexanone	541-85-5	9.60	0.31	1.04	3.55	1069	1060	
Ethyl 3-methylbutanoate	108-64-5	9.62	0.41	1.06	3.32	1069	1056	✓
2-Methyl-1-propanol	78-83-1	9.80	0.52	0.4	3.75	1080	1085	✓
Hexanal	66-25-1	9.86	0.12	0.86	1.26	1084	1088	✓
Sabinene	3387-41-5	10.80	0.19	1.51	2.47	1114	1115	✓
3-Penten-2-one	625-33-2	10.93	0.15	0.63	5.77	1119	1121	
1-Acetylcyclohexene	932-68-1	11.00	0.19	0.95	4.19	1121	ND	
2,3-Hexanedione	3848-24-6	11.07	0.19	0.69	4.04	1123	1136	✓
Ethylbenzene	100-41-4	11.40	0.26	0.89	3.08	1135	1125	✓
5-Methyl-2-hexanone	110-12-3	11.40	0.2	1.01	7.69	1135	1150	✓
3-Isopropyl-6-methyl-1-cyclohexene	591-49-1	11.60	0.37	1.71	3.48	1141	ND	
δ-Carene	13466-78-9	11.60	0.16	1.59	3.11	1141	1149	✓
Propyl butanoate	105-66-8	11.61	0.32	1.14	3.26	1144	1153	✓
1-Butanol	71-36-3	11.71	0.18	0.41	2.87	1146	1150	✓
2,3-Heptanedione	96-04-8	11.73	0.4	0.82	4.55	1146	1153	✓
Ethyl pentanoate	539-82-2	11.73	0.21	1.14	3.1	1146	1139	✓
β-Myrcene	123-35-3	12.00	0.36	1.37	7.52	1155	1150	✓
α-Phellandrene	99-83-2	12.07	0.27	1.5	13.01	1157	1160	✓
Butyl isobutanoate	97-86-9	12.17	0.42	1.43	2.55	1158	1149	
Propyl 3-methylbutanoate	557-00-6	12.21	0.45	1.38	2.89	1164	1145	✓
Pyridine	110-86-1	12.40	1.24	0.54	7.61	1169	1183	✓
α-Terpinene	99-86-5	12.53	0.06	1.52	3.72	1173	1170	
2-Heptanone	110-43-0	12.55	0.15	1.04	2.24	1175	1173	✓
Heptanal	111-71-7	12.73	0.73	1.05	6.56	1180	1184	✓
Cyclopentanone	120-92-3	12.73	0.24	0.72	11.99	1180	1180	✓
(E)-3-Methyl-2-butenal	497-03-0	13.07	0.06	0.67	10.13	1190	1200	✓
Limonene	138-86-3	13.07	0.07	1.51	1.22	1190	1210	✓
2-Methyl-1-butanol	137-32-6	13.13	0.06	0.48	1.78	1192	1193	✓
Dodecane	112-40-3	13.23	1	3.38	8.66	1200	1200	✓
β-Phellandrene	555-10-2	13.40	0.01	1.52	4.38	1200	1200	✓
Ethyl 4-methylpentanoate	7452-79-1	13.49	0.42	1.35	3.39	1204	1204	✓
(E)-2-Hexenal	6728-26-3	13.93	0.17	0.84	5.62	1218	1216	
2-Pentylfuran	3777-69-3	14.13	0.18	1.13	2.17	1222	1228	✓
Methylpropyl disulfide	2179-60-4	14.13	0.07	0.98	1.45	1222	1230	✓
6-Methyl-2-heptanone	928-68-7	14.33	0.26	1.18	7.71	1228	1228	✓
1-Pentanol	71-41-0	14.47	0.06	0.48	2.14	1232	1250	✓
(E)-4-Heptenal	6739-80-8	14.47	0.1	0.88	4.76	1232	1243	
3-Methyl-3-buten-1-ol	763-32-6	14.53	0.22	0.43	7.85	1234	1235	✓
γ-Terpinene	99-85-4	14.53	0.14	1.47	9.41	1234	1250	✓
Butyl butanoate	109-21-7	14.55	0.33	1.41	2.41	1234	1245	✓
3,4-Dimethylthiophene	632-13-9	14.80	0.45	0.8	2.89	1242	1253	
5-Methyl-2-heptanone	110-12-3	14.87	0.57	1.18	1.94	1244	1252	✓
3-Octanone	106-68-3	14.87	0.15	1.26	3.2	1244	1244	✓
Butyl 2-methylbutanoate	15706-73-7	14.91	0.15	1.67	2.44	1245	1219	
Styrene	100-42-5	14.93	0.17	0.74	9.07	1246	1260	✓
Ethyl hexanoate	123-66-0	14.95	0.39	1.33	2.99	1246	1246	✓
cis-β-Ocimene	3338-55-4	15.01	0.29	1.39	5.94	1251	1240	
3-Methyl-1-butyl butanoate	106-27-4	15.39	0.27	1.6	2.29	1258	1255	✓
o-Cymene	527-84-4	15.40	1.04	1.21	5.76	1259	1268	✓
Allyl methyl disulfide	2179-58-0	15.67	0.07	0.81	4.58	1267	1266	✓
2-Ethyltoluene	611-14-3	15.73	0.2	1.05	1.44	1269	1260	✓
Terpinolene	586-62-9	15.73	0.7	1.47	18.18	1269	1280	✓
2-Octanone	111-13-7	15.80	0.05	1.2	1.97	1271	1287	✓
3-Hydroxy-2-butanone	51555-24-9	15.98	0.18	0.41	2.67	1277	1271	✓
Octanal	124-13-0	16.00	0.27	1.21	2.74	1280	1295	✓
n-Pentyl butanoate	540-18-1	16.02	0.34	1.57	3.57	1280	1305	
1-Octen-3-one	4312-99-6	16.33	0.33	1.04	5.74	1287	1298	✓
(E)-3-Hepten-2-one	14686-13-6	16.33	0.23	0.94	12.43	1287	1274	
2-Heptanol	543-49-7	16.73	0.21	0.67	2.11	1299	1298	✓
Tridecane	629-50-5	16.20	0.28	3.57	4.33	1300	1300	✓
2-Methyl-2-buten-4-ol	115-18-4	16.80	0.3	0.42	4.98	1301	1311	
2,5-Dimethylpyrazine	123-32-0	17.07	0.11	0.69	5.63	1309	1320	
(E)-2-Heptenal	18829-55-5	17.13	0.37	0.96	2.47	1311	1310	✓
6-Methyl-5-hepten-2-one	110-93-0	17.53	0.09	0.97	7.98	1323	1323	✓
1-Hexanol	111-27-3	17.80	0.29	0.55	2.86	1331	1340	✓
1-Ethenyl-2-methyl benzene	611-15-4	18.20	0.23	0.85	6.79	1343	1342	
Allyl Isothiocyanate	57-06-7	18.20	0.07	0.61	0.95	1343	1356	✓
2-Hydroxy-3-pentanone	600-46-8	18.33	0.08	0.49	2.3	1347	1360	
Dimethyl trisulfide	3658-80-8	18.87	1.58	0.79	2.95	1363	1362	✓
2-Nonanone	821-55-6	19.13	0.36	1.31	8.68	1371	1370	✓
2-Furancarbonitrile	532-13-2	19.33	0	0.45	1.8	1377	ND	
Trimethylpyrazine	14667-55-1	19.47	0.05	0.79	3	1381	1389	
3-Octen-2-one	1669-44-9	19.73	0.39	1.05	1.19	1388	1410	✓
2-Octanol	123-96-6	19.93	0.12	0.75	7.84	1394	1400	✓
Nonanal	124-19-6	19.33	0.64	1.33	2.55	1397	1395	✓
Tetradecane	629-59-4	19.40	0.29	3.65	7.23	1400	1400	✓
Perillene	18457-81-1	20.20	0.13	1.02	1.67	1415	1430	
(E)-2-Octenal	2548-87-0	20.47	0.12	1.07	14.02	1415	1430	✓
p-Cymenene	1195-32-0	20.67	0.34	0.96	3.57	1416	1420	✓
4-Methoxytoluene	104-93-8	20.73	0.2	0.77	1.91	1418	1430	✓
1-Octen-3-ol	3391-86-4	20.93	0.03	0.63	3.65	1424	1430	✓
1-Heptanol	111-70-6	21.07	0.16	0.61	3.05	1428	1440	✓
3-Butenyl isothiocyanate	4426-95-5	21.27	0.11	0.73	4.54	1434	1459	
2-Pentylthiophene	4861-58-9	21.33	0.07	1.21	4.84	1436	1438	
α-Cubebene	17699-14-8	21.40	0.75	2.44	4.13	1438	1440	✓
Furfural	98-01-1	21.60	0.27	0.42	5.56	1444	1450	✓
Methional	3268-49-3	21.27	0.35	0.58	2.36	1446	1454	✓
Tetramethylpyrazine	1124-11-4	21.67	0.62	0.88	1.82	1446	1460	
Acetic acid	64-19-7	21.80	0.19	0.19	4.85	1450	1450	✓
δ-Elemene	20307-84-0	21.80	0.15	2.23	9.69	1450	1460	✓
2-Propyl-1-pentanol	10042-59-8	22.20	0.21	0.7	1.78	1464	ND	
α-Copaene	3856-25-5	22.20	0.1	2.47	3.5	1464	1480	
Ethyl octanoate	106-32-1	22.27	0.13	2.13	1.67	1466	1463	
(E)-1-Butenylbenzene	766-90-5	22.34	0.6	0.96	6.42	1468	1479	
(E,E)-2,4-Heptadienal	05/03/4313	22.47	0.65	0.76	1.34	1474	1474	
2-Decanone	693-54-9	22.47	0.29	1.4	2.4	1474	1480	
Decanal	112-31-2	22.60	0.09	1.43	2.43	1479	1480	✓
2-Acetylfuran	1192-62-7	22.87	0.29	0.51	10.95	1488	1488	✓
3-Nonen-2-one	6410-44-6	23.00	0.09	1.13	1.8	1493	1515	
2-Nonanol	628-99-9	23.07	0.43	0.83	5.19	1495	1505	
Pentadecane	629-62-9	23.18	0.46	3.66	3.9	1500	1500	✓
Pyrrole	109-97-7	23.20	0.05	0.3	7.2	1500	1500	✓
Benzaldehyde	100-52-7	23.47	0.15	0.59	8.38	1510	1508	✓
Sesquiterpenoid 2	ND	23.47	0.45	2.29	14	1510	ND	
(E)-2-Nonenal	18829-56-6	23.73	0.92	1.17	1.68	1519	1532	✓
Sesquiterpenoid 3	ND	23.93	0.48	2.24	6.59	1526	ND	
Linalool	78-70-6	24.00	1	0.76	5.13	1529	1530	✓
1-Octanol	111-87-5	24.20	0.67	0.69	7.29	1536	1534	✓
Cyclohexanemethanol	823-59-6	24.33	0.22	0.59	1.62	1541	ND	
Tridecanoic acid	638-53-9	24.33	0.32	0.23	3.26	1541	1530	
Propanoic acid	79-09-4	24.33	0.32	0.23	3.26	1541	1530	✓
Sesquiterpenoid 4	ND	24.33	0.41	2.22	2.09	1541	ND	
2,3-Butanediol	513-85-9	24.73	0.23	0.31	1.54	1555	1542	
α-Bergamotene	17699-05-7	24.80	1.05	2.1	3.26	1557	1570	✓
5-Methyl-2-furfural	620-02-0	24.93	0.33	0.54	13.93	1562	1562	✓
Sesquiterpenoid 6	ND	25.07	0.65	2.13	6	1567	ND	
α-Bisabolene	17627-44-0	25.33	0.34	2.14	6.3	1576	1570	
2-Methylpropanoic acid	79-31-2	25.40	0.55	0.25	3.92	1579	1587	✓
(E)-6-Methyl-3,5-heptadien-2-one	1604-28-0	25.40	0.09	0.83	2.85	1579	1582	
4-Cyclopentene-1,3-dione	4655-45-4	25.40	0.34	0.44	5.5	1579	1605	
β-Elemene	515-13-9	25.47	0.05	1.88	5.76	1581	1580	
Sesquiterpenoid 5	ND	25.47	0.32	2.18	3.95	1581	ND	
3,5,5-trimethyl-2-Cyclohexen-1-one	78-59-1	25.53	0.19	1	3.07	1583	1576	
2-Undecanone	112-12-9	25.60	0.86	1.47	2.49	1586	1580	✓
Undecanal	112-44-7	25.80	0.23	1.5	2.59	1593	1606	✓
Sesquiterpenoid 7	ND	25.93	0.14	2.1	2.95	1598	ND	
Hexadecane	544-76-3	26.01	0.17	3.62	6.24	1600	1600	✓
Benzonitrile	100-47-0	26.09	0.26	0.56	7	1600	1583	✓
2-Acetyl-5-methylfuran	1193-79-9	26.13	0.08	0.61	3.06	1605	1603	✓
2-(2-Ethoxyethoxy)ethanol	111-90-0	26.20	0.41	0.5	1.47	1607	1615	✓
Sesquiterpenoid 8	ND	26.20	0.37	2.06	2.94	1607	ND	
2-Decanol	112-30-1	26.22	0.56	0.89	3.88	1609	1621	
Aromadendrene	489-39-4	26.47	0.43	2.09	3.41	1617	1620	
Menthol	2216-51-5	26.80	0.8	0.84	4.69	1629	1630	✓
Butanoic acid	107-92-6	26.87	0.26	0.26	3.54	1631	1622	✓
γ-Elemene	29873-99-2	26.87	0.33	1.81	2.59	1631	1633	
β-Terpinyl acetate	80-26-2	28.92	0.08	1.24	3.3	1633	1622	
Benzeneacetaldehyde	122-78-1	27.00	0.13	0.6	6.19	1636	1636	✓
Acetophenone	98-86-2	27.20	0.49	0.64	6.6	1643	1623	✓
Sesquiterpenoid 9	ND	27.20	0	1.95	9.19	1643	ND	
1-Nonanol	143-08-8	27.27	0.2	0.74	4.25	1645	1640	✓
2-Furanmethanol	98-00-0	27.33	0.14	0.3	4.04	1648	1635	
Sesquiterpenoid 10	ND	27.40	0.17	1.95	4.36	1650	ND	
β-Farnesene	18794-84-8	27.53	0.33	1.75	2.41	1655	1660	✓
Sesquiterpenoid 11	ND	27.53	0.52	1.99	8.5	1655	ND	
α-Caryophyllene	6753-98-6	27.87	0.37	1.93	8.69	1667	1665	✓
2-Methylbutanoic acid	116-53-0	28.20	0.05	0.28	1.99	1679	1670	✓
Sesquiterpenoid 12	ND	28.40	0.17	1.88	2.13	1686	ND	
γ-Caprolactone	695-06-7	28.58	0.15	0.69	5.19	1692	1694	✓
(E,E)-2,4-Nonadienal	5910-87-2	28.60	0.2	0.92	4.93	1693	1690	
2-Dodecanone	6175-49-1	28.67	0.45	1.53	5.18	1695	1704	✓
Heptadecane	629-78-7	28.73	0.26	3.58	3.34	1700	1700	✓
3-(Methylthio)-1-propanol	505-10-2	28.87	0.19	0.42	2.31	1703	1710	✓
Dodecanal	112-54-9	28.87	0.21	1.56	2.35	1703	1716	✓
2-Undecanol	1653-30-1	28.93	0.05	0.97	2.11	1705	1706	✓
Sesquiterpenoid 13	ND	28.93	0.15	1.89	2.37	1705	ND	
3-Undecen-2-one	10522-37-9	29.20	0.35	1.27	11.56	1715	1710	
β-Bisabolene	495-61-4	29.33	0.06	1.76	8.25	1719	1715	
Phenylacetone	7624-24-0	29.40	0.51	0.68	3.84	1722	1710	
Acetamide	60-35-5	29.73	0.51	0.26	3.14	1734	1748	✓
Pentanoic acid	109-52-4	30.07	0.23	0.28	7.32	1746	1744	✓
1-Decanol	112-30-1	30.13	0.21	0.8	4.83	1748	1750	✓
Sesquiterpenoid 1	ND	30.20	0.47	1.74	5.46	1750	ND	
Methyl salicylate	119-36-8	30.37	0.62	0.66	4.19	1762	1747	✓
1,1,6-Trimethyl-1,2-dihydro naphthalene	30364-38-6	30.37	0.24	1.3	2.81	1765	1751	
p-Methylacetophenone	122-00-9	30.38	1.18	0.72	6.78	1767	1751	
α-Curcumene	644-30-4	30.67	0.37	1.45	10.89	1767	1770	
Sesquiterpenoid 16	ND	30.67	0.46	1.67	4.57	1767	ND	
Sesquiterpenoid 14	ND	30.67	0.46	1.67	4.57	1767	ND	
Ethyl 2-phenylacetate	101-97-3	30.89	0.2	0.77	4.93	1771	1768	
4-(1-Methylethyl)-benzaldehyde	122-03-2	30.93	0.27	0.83	10.96	1776	1770	✓
Sesquiterpenoid 17	ND	30.93	0.39	1.57	2.99	1776	ND	
Sesquiterpenoid 15	ND	30.93	0.39	1.57	2.99	1776	ND	
2-(2-Butoxyethoxy)ethanol	112-34-5	31.13	0.22	0.61	5.19	1784	1796	
δ-Valerolactone	542-28-9	31.13	0.06	0.68	11.55	1784	1780	
Octadecane	593-45-3	31.33	0.32	3.53	8.34	1798	1800	✓
(E,E)-2,4-Decadienal	25152-84-5	31.60	0.07	0.98	2.07	1800	1800	
2-Tridecanone	593-08-8	31.60	0.19	1.58	4.14	1800	1814	
Tridecanal	10486-19-8	31.80	0.09	1.6	2.13	1807	1810	✓
1-Phenyl-2-propanol	98-85-1	31.87	0.03	0.53	1.13	1810	1778	
Phenethyl acetate	103-45-7	31.87	0.11	0.73	5.34	1810	1820	
Anethole	104-46-1	32.07	0	0.76	2.06	1817	1820	✓
4-Methyl-pentanoic acid	646-07-1	32.33	1.12	0.28	7.53	1827	1817	✓
(E)-Calamenene	7299-79-8	32.33	0.25	1.49	14.11	1827	1820	
(5E)-6,10-Dimethyl-5,9-undecadien-2-one	3796-70-1	32.80	0.18	1.23	0.71	1843	1864	
3-Phenylfuran	700-33-0	32.80	0.22	0.61	1.71	1843	1839	
Butyl benzoate	136-60-7	33.13	0.27	0.93	2.22	1855	1871	
Ethyl 3-phenylpropanoate	2021-28-5	34.02	0.21	0.84	5.75	1872	1872	
2-Tridecanol	1653-31-2	34.33	0.25	1.1	1.99	1898	1898	
Butyl hydroxytoluene	128-37-0	34.33	0.95	1.19	0.04	1898	1898	
2-Tetradecanone	2345-28-0	34.33	0.08	1.63	2.62	1898	1900	
Tetradecanal	124-25-4	34.60	0.45	1.65	1.98	1908	1910	✓
2-Phenyl-2-butenal	104-67-6	34.73	0.09	0.7	3.38	1912	1922	
5-Methylhexanoic acid	628-46-6	35.07	0.37	0.3	1.67	1924	1914	
1-Dodecanol	112-53-8	35.53	0.14	0.92	7.26	1941	1930	✓
Heptanoic acid	111-14-8	35.87	0.19	0.32	7.04	1953	1950	✓
Phenol	108-95-2	36.53	0.76	0.29	15.38	1990	1987	✓
2-Pentadecanone	2345-29-1	37.00	0.27	1.67	3.92	2011	2021	
Pentadecanal	09/11/2765	37.20	0.42	1.7	2.55	2020	2030	
γ-Nonalactone	104-61-0	37.27	0.11	0.87	4.06	2023	2023	
3-Methylphenol	108-39-4	38.33	0.66	0.33	3.22	2072	2081	✓
Octanoic acid	124-07-2	38.67	0.12	0.34	1.5	2083	2070	✓
Hexadecanal	629-80-1	38.60	0.08	1.76	1.71	2084	2100	✓
1-Tetradecanol	112-72-1	40.47	0.4	1.03	3.83	2169	2173	
4-Ethylphenol	123-07-9	40.53	0.03	0.35	0.53	2172	2170	✓
p-Cresol	106-44-5	41.00	0.21	0.27	3.1	2194	2180	✓
2-Acetylaniline	614-93-1	41.60	0.17	0.47	2.83	2196	2202	
Nonanoic acid	112-05-0	41.40	0.11	0.35	4.66	2212	2211	✓
4-Isopropyl-1,6-dimethylnaphthalene	611-13-2	41.80	0.03	1.02	7.46	2230	2220	
Methylethylmaleimide	33324-52-4	42.80	0.52	0.37	4.61	2255	2260	
Ethyl hexadecanoate	628-97-7	42.53	0.33	1.9	5.77	2264	2250	
1-Pentadecanol	629-76-5	42.80	0.18	1.08	2.84	2276	2254	
Decanoic acid	334-48-5	43.08	0.2	0.38	1.12	2291	2280	✓
4-Methyl-5-(2-hydroxyethyl)thiazole	137-00-8	43.33	0.29	0.41	3.49	2300	2311	
4-Ethylphenol	123-07-9	43.60	0.07	0.57	2.81	2312	2315	✓
2,4-Di-tert-butylphenol	96-76-4	43.60	0.07	0.57	2.81	2312	2315	
Octadecanal	638-66-4	44.53	0.3	1.79	3.67	2355	2350	✓
1-Hexadecanol	36653-82-4	45.07	0.16	1.13	2.07	2379	2365	✓
γ-Dodecalactone	07/05/2305	45.13	0.22	1.01	3.67	2382	2381	
Indole	120-72-9	46.40	0.32	0.38	5.31	2440	2441	✓
5-Methylindole	120-71-8	47.40	0.31	0.43	3.96	2486	2468	
Hexanamide	628-95-9	48.20	0.13	0.55	4.05	2522	2520	
Hexanoic acid	142-62-1	48.33	0.13	0.43	4.77	2528	2523	✓
Dodecanoic acid	143-07-7	48.33	0.13	0.43	4.77	2528	2523	✓
Decyl decanoate	7299-14-3	50.67	0	0.7	4.76	2561	2565	
Hydrocinnamic acid	501-52-0	51.27	0.29	0.25	5.65	2648	2638	
(Z)-9-Octadecenal	509-30-4	51.41	0.07	1.69	2.46	2685	2693	
1,3-Dihydro-(2H)-indol-2-one	59-48-3	54.07	0.27	0.39	2.65	2790	ND	✓

The identified VOCs covered a broad range of chemical classes and reflect the diverse metabolites of the gut microbiota. Among these, short-chain fatty acids (SCFAs) such as acetic, butanoic, and pentanoic acids were particularly abundant, together with 34 alcohols, 35 aldehydes, 40 ketones, 24 esters, 20 aromatic compounds, 15 heterocyclic compounds, 12 hydrocarbons, 8 nitrogen-containing compounds, 5 sulfides, 4 lactones, 3 pyrazines, 2 isothiocyanates, and 50 terpenes (see Supplementary Table 2 – ST2). This distribution is consistent with previous fecal volatilome profiles obtained using 1D-GC, which reported broad sets of VOCs (441 in total [11]) including acids, aldehydes, ketones, sulfur-containing compounds, and microbial aromatics such as indole and *p*-cresol. In addition, several newly detected compounds expanded the known fecal volatilome, including unsaturated aldehydes and ketones (e.g., (E)−2-hexenal, (E,E)−2,4-heptadienal), long-chain alcohols and esters (e.g., 1-pentadecanol, ethyl 2-phenylacetate), and aromatic or heterocyclic derivatives such as 2,5-dimethylpyrazine and 3,4-dimethylthiophene. Terpenoids such as α-bisabolene, β-terpinyl acetate, and perillene were also observed, along with several aliphatic hydrocarbons and cyclic carbonyls. These newly identified VOCs represent lipid oxidation products, microbial fermentation derivatives, plant specialized metabolites (e.g., terpenes), and amino acid catabolites, further highlighting the chemical diversity and metabolic complexity of the fecal volatile profile (see Table 1, which also specifies whether compounds were previously reported in the literature as characteristic fecal VOCs) [25, 38].

The present GC×GC-TOFMS approach not only confirms those major chemical classes but also reveals additional minor constituents (e.g., terpenoids) that might be overlooked by less powerful techniques. Many of these volatiles have clear biological origins and relevance: SCFAs and their esters arise from bacterial fermentation of dietary fibers, indole and phenol derivatives from tryptophan and tyrosine metabolism, various alcohols and aldehydes from microbial or host metabolic processes [17, 25, 27]. The consistent detection of such compounds across subjects suggests a core volatilome, shared by most individuals [25]. This core set includes key microbial metabolites (e.g., indole, a quorum signaling molecule associated with *E. coli*, gut barrier function and, *p-*cresol, a tyrosine-derived phenol) known to influence host physiology [24]. The broad presence of alcohols, acids, esters, and sulfur compounds in our dataset is also in line with other metabolomic studies, which note that fecal VOC profiles are predominantly composed of oxygenated metabolites (carboxylic acids, alcohols, esters, ketones) along with sulfur- and nitrogen-containing volatiles. Such chemical richness underscores the complex interplay of gut microbial metabolism and host digestion. Targeted fingerprinting of the volatilome, focusing on known or biologically significant VOCs, also highlighted specific compounds and classes that distinguish the experimental conditions. In particular, SCFA esters (esters of propanoic and butanoic acids) emerged as important features, along with several primary alcohols, aldehydes, and terpenoids, that had the highest informational value in discriminating the results of the dietary intervention. These targeted compounds were not only statistically significant in chemometric models but are also biologically plausible markers: ethyl butanoate and propanoate, for example, may indicate shifts in bacterial fermentation pathways or changes in host lipid metabolism under the probiotic and diet [39–41]. Similarly, changes in certain terpenoids could reflect alterations in diet-derived plant secondary metabolites or their microbial modifications. The enrichment of butanoate and propanoate ester signals alongside shifts in alcohols and aldehydes is consistent with metabolic changes expected with a gluten-free diet and probiotic supplementation (e.g., increased fiber fermentation leading to higher SCFA production, which can form volatile esters).

**Fig. 3:**
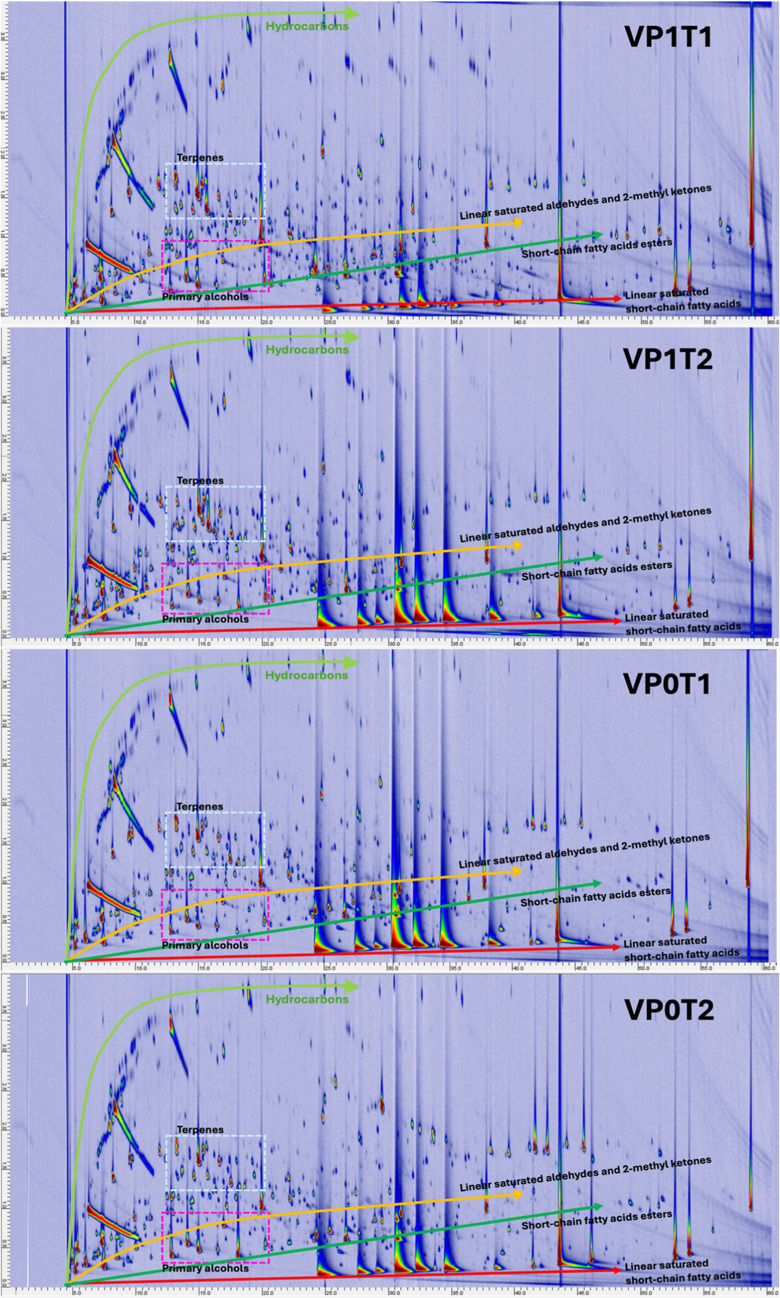
Contour plots of GC×GC-TOF MS total ion current (TIC) for four representative fecal samples: VP1T1, VP1T2, VP0T1, and VP0T2. The ^1^*t*_*R*_ (*x*-axis) and ^2^*t*_*R*_ (*y*-axis) are shown, with a color scale indicating signal intensity. Colored arrows and lines indicate typical elution regions of key volatile classes—hydrocarbons, terpenoids, primary alcohols, short-chain fatty acids and their esters, linear aldehydes, and 2-methyl ketones

The set of GC×GC-TOFMS total ion current (TIC) contour plots in Fig. 3 provides a comprehensive representation of the fecal volatilome across representative samples from the four experimental groups. Chromatographic separation was achieved by adopting a polar primary column (100% PEG) and a mid-polar secondary phase (OV1701). Compounds are separated along the ^1^D according to their relative polarity/volatility (prevalence of hydrogen bonding and dipole-dipole forces) and along the ^2^D mainly by volatility (dominance of dispersive interactions, e.g., Van der Waals and π-π [42]). This bidimensional architecture allows enhanced resolution of structurally and functionally related metabolites and facilitates their classification by chemical functionality. The resulting chemical space is characterized by reproducible elution domains, with distinct regions corresponding to specific classes of volatiles. Hydrocarbons and non-polar terpenoids elute early along the ^1^D while result highly retained on the ^2^D, whereas SCFAs (e.g., acetic, propanoic, butanoic acids) are highly retained along the ^1^D without any substantial interaction with the ^2^D at their actual elution temperature from the system. Their corresponding esters appear as a contiguous but more dispersed band in the middle region of the chromatographic space overlapped with medium-to-polar functionalities, e.g., primary alcohols, saturated and unsaturated aldehydes, ketones, and lactones. Homologous series of aliphatic aldehydes, alcohols, and methyl ketones display diagonal or banded elution patterns, with progression in carbon number correlating with increasing ^1^*t*_*R*_ values and subtle shifts along ^2^*t*_*R*_. Aromatic and heterocyclic volatiles elute in later retention windows, consistent with their higher molecular weight and greater polarity. Despite interindividual and treatment-related variability in compound intensities and signal complexity, the overall distribution and spatial organization of the contour plots remain conserved across conditions, highlighting the presence of a shared fecal volatilome core. This includes abundant oxygenated metabolites (carboxylic acids, alcohols, esters, and ketones), as well as microbially derived sulfur- and nitrogen-containing compounds. The chromatographic structure of each sample provides high-density information suitable for both untargeted and targeted fingerprinting approaches, enabling consistent chemical classification and facilitating inter-group comparisons.

### Overview of VOC signatures across groups and dietary intervention

**Fig. 4:**
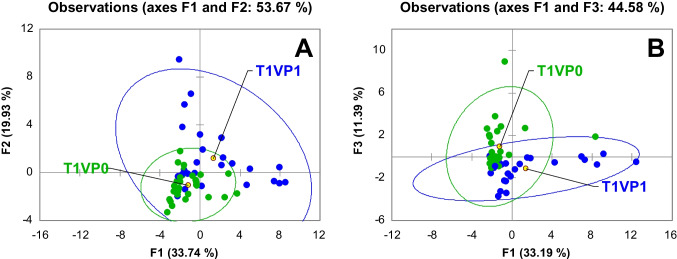
PCA on untargeted and targeted (UT) peak features (% responses); ellipses set at 95% confidence level, showing probiotic (VP1) vs. placebo (VP0) natural clustering. **A** All 35 Fisher‐filtered UT features (*F* > 6 on % normalized responses) at T1 (after 4 weeks of gluten withdrawal) were included. VP0 and VP1 samples overlap within their confidence ellipses, although VP1 exhibits greater dispersion along both PC 1 and PC 2, indicating incipient shifts in SCFA esters and terpenoid metabolites. **B** PCA on the same 35 discriminant features but using absolute peak areas (*F* > 5). Here, VP1 samples shift toward higher PC 1 scores and VP0 toward lower PC 1, with partially overlapping 95% ellipses—demonstrating that raw abundances of key VOCs differ between arms even when normalized profiles appear similar

A first unsupervised exploration of the fecal volatilome was conducted by principal component analysis (PCA) considering the full set of features (% response) obtained by GC×GC-TOFMS. Results are illustrated in Fig. 4A. At baseline (T1; *n* = 81 analyses), a total of 35 peak-region features (peak areas filtered by Fisher discriminant ratio *F* > 6 on percent normalized responses) were retained for PCA. As shown in Fig. 4A, the combined PC1 (33.74% of total variance) and PC2 (19.93%) account for 53.67% of the explained variance. Samples from the probiotic (VP1T1; blue indicators) and placebo (VP0T1; green indicators) arms largely overlap within a 95% confidence ellipse, suggesting that, after 4 weeks of a gluten-free diet, the overall VOC signature remains similar between groups. However, individuals receiving probiotics exhibited slightly greater dispersion along PC1, indicating early compositional shifts in specific subgroups of volatiles (e.g., SCFA esters and terpenoids), whereas placebo samples clustered more tightly (smaller internal variability). This overlap in % response space reflects the normalizing effect of total ion count scaling, which can mask absolute differences in VOC abundance. To avoid possible masking of absolute abundance differences by normalization, the same feature set was re‐evaluated using the normalized peak areas (absolute responses vs. ISs) after the Fisher threshold had been lowered to *F* > 5 (*n* = 35). In the corresponding plot (Fig. 4B), PC1 and PC3 together explain 44.58% of the variance; VP1T1 samples shift towards positive PC1, while VP0T1 samples cluster towards negative PC1. The confidence ellipses still partially overlap, but the increased spread of VP1T1 along PC1 and PC2 confirms that the absolute abundances of the major metabolites differ under probiotic supplementation, even when normalized profiles appear similar. This observation suggests that probiotic administration modulates microbial fermentation and oxidation processes—reflected in increased SCFA esters and an early increase in aromatic hydrocarbons—before gluten is reintroduced.

**Fig. 5:**
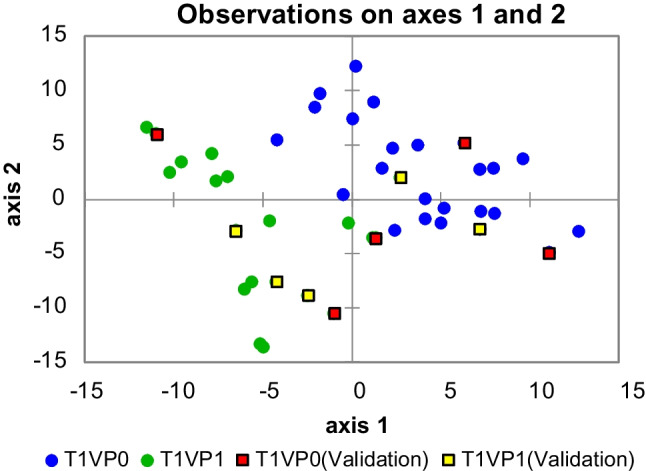
Partial least squares discriminant analysis (PLS-DA) on untargeted and targeted (UT) peak features (% responses) at T1 (4 weeks of gluten withdrawal). Blue circles represent placebo samples (VP0T1), green circles represent probiotic samples (VP1T1), red squares denote VP0T1 validation samples, and yellow squares denote VP1T1 validation samples. The model was built using 35 Fisher‐filtered features (*F* > 6) and yields 89% cross‐validated classification accuracy. Samples separate primarily along latent variable 1 (LV1), with VP0T1 projecting to negative LV1 scores and VP1T1 to positive LV1 scores. Ellipses denote 95% confidence intervals for each treatment group. Major VIP discriminants include short‐chain fatty acid esters (e.g., butanoic acid and propanoic acid derived esters), aromatic hydrocarbons (o-xylene; 1,3-dimethylbenzene), and terpenoids (β-myrcene; γ-terpinene)

Building on these unsupervised results, a supervised classification by PLS-DA was conducted at T1 using both percent‐normalized and absolute area-based data matrices (each *n* = 35 features). For the PLS-DA on percent responses (Fig. 5), three latent variables were retained, accounting for 41.6% of the *X*-variance and 63.2% of the *Y*-variance (*R*^2^*X*(cum) = 0.416; *R*^2^*Y*(cum) = 0.632), with a cumulative predictive ability *Q*^2^(cum) of 0.388. According to the Monte Carlo cross-validation strategy, this model achieved a mean cross-validated classification accuracy of 89%. In this context, the calibration set (*n* = 81) yielded 90.2% correct classifications (41/45 VP0 and 48/43 VP1 correctly assigned), while all samples in the external validation set were correctly predicted. Consistently with this performance, most VP0T1 samples project to negative LV1 scores, whereas VP1T1 samples cluster on the positive side of LV1, while the validation samples (square markers) fall within the corresponding class domains. Variables showing variable importance in projection (VIP) greater than 1.5 include SCFA esters (butanoic and propanoic acid derivatives), aromatics (o-xylene; 1,3-dimethylbenzene), and terpenoids (β-myrcene), indicating early probiotic‐induced modulation of esterification pathways and terpene biotransformations. In the absolute‐area PLS-DA, the classification accuracy increases slightly to 90%; the VIPs again include SCFA esters and aldehydes (e.g., heptanal; nonanal), suggesting that probiotics may influence microbial oxidoreductive enzyme systems (e.g., alcohol dehydrogenases; lipoxygenases) even in the absence of gluten.

PCA was also performed on the mean-centered data matrix on samples collected 2 weeks after gluten reintroduction (T2; shown in Supplementary Fig. 2 – SF2); the VOCs matrix comprises 25 Fisher‐filtered features. The first two principal components explain 33.36% (PC1) and 14.80% (PC2) of the total variance, respectively—together accounting for 51.16% of the variance. In this unsupervised projection, samples from probiotic‐treated subjects (VP1T2) tend to score on the positive side of PC1, while VP0T2 tends to score on the negative side. However, their 95% confidence regions still overlap, indicating that gluten reintroduction increases intra-group heterogeneity, especially among VP1T2, so that PCA can no longer resolve two independent clusters.

Attempts to create a supervised PLS-DA classifier on the same data at T2 did not achieve acceptable cross-validated accuracy; the validation samples did not reliably separate by treatment. Although VP1T2 samples have on average higher levels of short-chain fatty acid esters (e.g., propanoic acid butyl ester; butanoic acid butyl ester), aromatic hydrocarbons (e.g., o-xylene; 1,3-dimethylbenzene), terpenoids (*e.g*., β-myrcene; γ-terpinene), lipid-derived alcohols (*e.g*., 1-hexanol; 1-octen-3-ol), and aldehydes (*e.g*., heptanal; nonanal), the increased interindividual variability under gluten challenge prevents consistent, linear discrimination. In other words, while probiotic supplementation continues to increase these VOC classes, their increased heterogeneity upon gluten reintroduction undermines both PCA clustering and PLS-DA classification.

In summary, gluten reintroduction amplifies fecal volatilome variability—particularly within the probiotic cohort—such that neither PCA nor PLS-DA provides robust classification distinguishing VP1T2 from VP0T2. Elevated SCFA esters, aromatics, and terpenoid compounds remain nominally higher in VP1T2, but their broader fluctuations under dietary challenge preclude reliable multivariate separation. Consequently, although probiotic‐induced metabolomic shifts are clear under gluten withdrawal, they become less reproducible once gluten is reintroduced, highlighting the dynamic, diet-dependent nature of gut microbial metabolism.

**Fig. 6:**
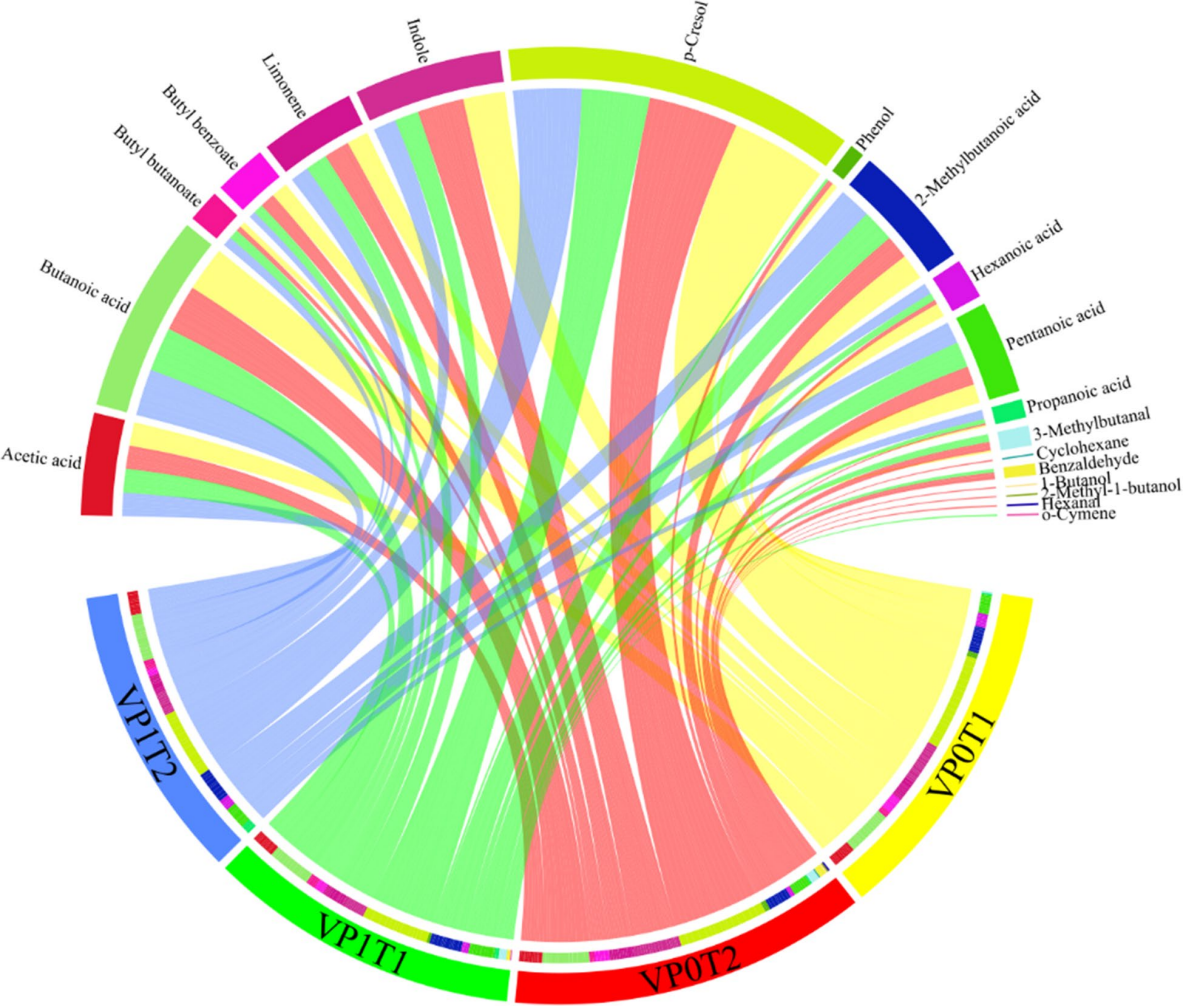
Chord diagram showing the mean % responses calculated for each metabolite–treatment arm; shifts in the volatilome profile for the different treatments are highlighted (see text for detailed comments)

The chord diagram of Fig. 6 visualizes the distribution and relative abundance of metabolites across the different treatment arms, providing an integrated overview of metabolomic shifts induced by the diet intervention. In this diagram, connections (chords) between treatment arms and metabolite sectors were drawn only when the metabolite’s percentage of the total normalized response exceeded 2%, with chord width proportional to the metabolite’s relative contribution within each treatment arm—thus reflecting the strength of association. The mean values calculated for each metabolite–treatment combination reveal clear metabolic trends: short-chain fatty acids displayed noticeable variability, with butanoic acid being higher in VP0T2 compared to VP1T1 and VP0T1, and 2-methylbutanoic acid reaching its peak in VP1T2, likely due to branched-chain amino acid catabolism [43]. Similarly, 3-methylbutanal, 1-octen-3-ol, and 2-methyl-1-butanol were more abundant in VP0T2, metabolites previously associated with irritable bowel syndrome (IBS) [44]. Distinct patterns also emerged among aromatic compounds, with indole consistently higher in VP0T2 and *p-*cresol elevated in both VP0T1 and VP0T2. Terpenes such as limonene and fatty acids including hexanoic and pentanoic acid also varied notably, showing their highest mean percentages in VP1T2.

### Differential effect of diet and probiotic intervention: insights and literature correlation

Gluten intake and dietary interventions (notably gluten withdrawal and probiotic supplementation) have been shown to significantly reshape the human fecal volatilome and metabolome by modulating gut microbial metabolism. Our dataset confirms the enrichment of short‐chain fatty‐acid esters after probiotic supplementation that Ferrocino et al. [28] observed in the same clinical cohort, including relative increases for ethyl, propyl, and butyl esters of butanoic and propanoic acids at both the four‐week gluten‐free stage and after gluten reintroduction. Similar ester‐dominated signatures were reported in healthy adults given a multistrain gluten‐degrading probiotic during escalating gluten doses [45] and in treated celiac children maintained on a gluten‐free diet for 2 years [46]. The convergence of three independent trials underscores ester formation as a robust metabolic read‐out of probiotic‐enhanced saccharolytic fermentation during gluten exposure or withdrawal. Moreover, our results show a concomitant attenuation of lipid‐derived aldehydes (e.g., heptanal, nonanal) and alcohols (e.g., 1‐octen‐3‐ol) in the probiotic arm during gluten reintroduction, fully mirroring the direction reported by Ferrocino et al. [28] and matching the decrease of identical VOC classes in treated vs. untreated celiac children reviewed by Di Cagno et al. [46]. The reproducibility of this pattern across age groups suggests that suppression of oxidative lipolysis products may represent an additional hallmark of a resilient gut ecosystem.

Moreover, our analysis revealed increased relative abundances of indole and γ-caprolactone in probiotic-treated individuals following gluten reintroduction. In contrast, Nikoloudaki et al*.* [47] reported an altered tryptophan metabolism characterized by increased fecal skatole (3-methyl-indole) coupled with decreased indole concentrations under their distinct probiotic formulation, indicating a shift from indole towards skatole production. Our analytical approach, employing GC×GC-TOFMS, provided comprehensive coverage compared to the one-dimensional SPME-GC-MS platform, thereby enhancing the detection of polar indolic compounds. Further supporting these findings, Ferrocino et al. observed decreased fecal indole levels that were directly associated with expansions of *Lactiplantibacillus plantarum* and *Bifidobacterium adolescentis* in participants receiving probiotics [23]. Taken together, these converging findings highlight that probiotic-driven modulation of tryptophan catabolism strongly depends on the specific microbial strains and underscore the critical role of analytical platform resolution in volatilomic assessments.

Supplementary Fig. 4 – SF4 shows box plots for indole, octanal, and γ-caprolactone at timepoint T1 (after 4 weeks of gluten withdrawal), comparing the VP1 and VP0 intervention groups. All three compounds display lower median signals in VP1 than in VP0, consistent with early probiotic effects: indole is reduced in the probiotic arm, suggesting partial suppression of proteolytic fermentation even before gluten reintroduction; octanal, a lipid oxidation marker, is diminished under probiotic treatment, reflecting lower oxidative stress, and γ‐caprolactone shows a marginal decrease in VP1, indicating subtle shifts in microbial lipid metabolism. In summary, emerging literature indicates that changes in fecal VOCs like indole, γ-caprolactone, and butanoic esters faithfully mirror the microbial and metabolic effects of gluten intake or withdrawal and probiotic interventions, supporting their utility as biomarkers of microbial activity and potentially guiding personalized dietary therapies [48]

### Tracking VOC signatures in a single individual: towards a personalized diet

The integration of untargeted-targeted fingerprinting with computer vision (CV)–based chromatographic comparison enables the visualization and interpretation of individual-specific volatilome profiles. This approach allows for the direct comparison of GC×GC-TOFMS contour plots between paired samples from the same subject, offering a qualitative tool for tracking changes induced by dietary interventions. In this context, comparative images were generated using a colorized fuzzy ratio algorithm, which calculates the pixel-wise difference between two aligned chromatograms. The resulting image highlights regions with a higher signal in the analyzed sample in green and regions with a higher signal in the reference sample in red. This visualization preserves the two-dimensional chromatographic space and provides an immediate overview of the most affected chemical domains. Notably, changes within key compositional regions—such as short-chain fatty acids (SCFAs), esters, primary alcohols, aldehydes, and terpenoids—can be observed in a single individual, in agreement with previously identified markers of intervention response.

**Fig. 7:**
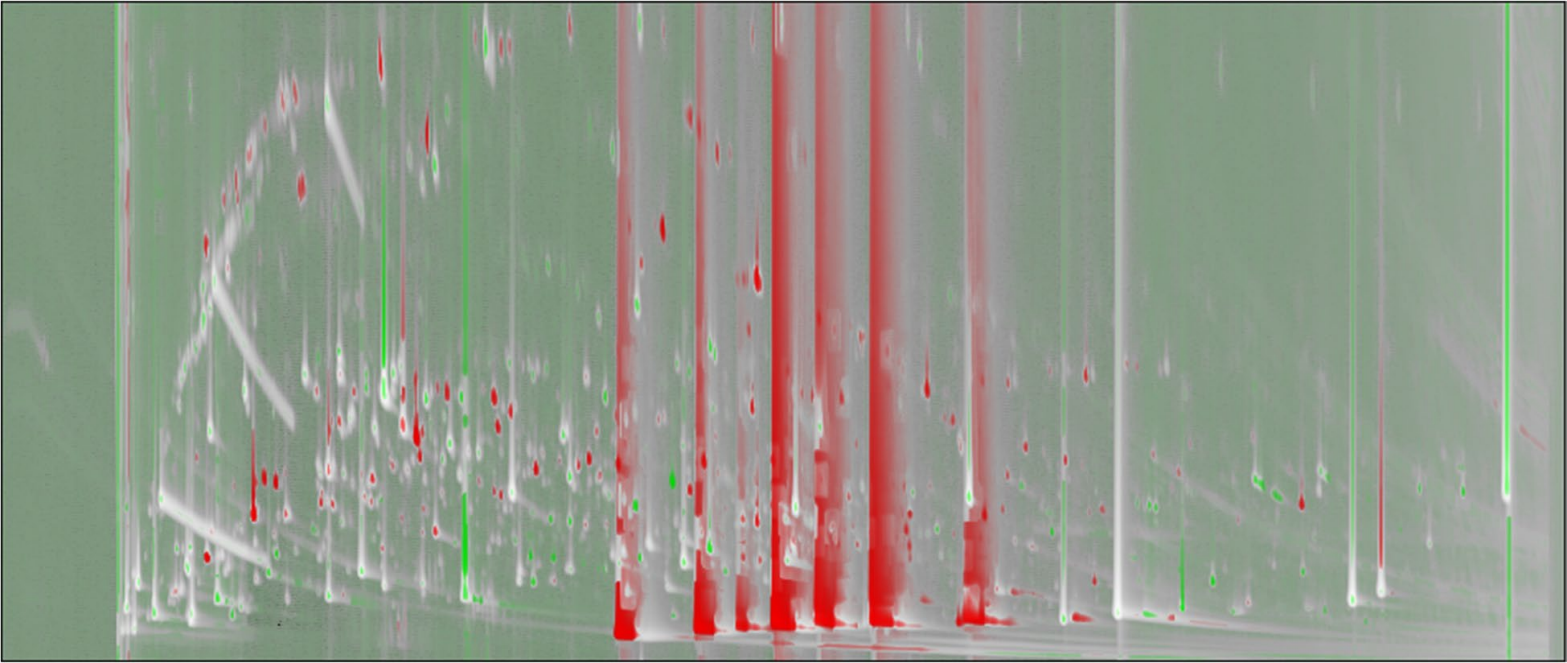
Computer vision-based comparison of GC×GC-TOFMS chromatograms from a single individual (VP1) at two timepoints (T0 vs. T1). The visualization is rendered as a colorized fuzzy ratio, where green areas indicate regions with higher signal intensity at T1 (after intervention) and red areas denote higher intensity at T0 (baseline)

As shown in Fig. 7, this CV-based visualization enables the identification of subject-specific alterations in VOC distribution patterns between experimental conditions (e.g., probiotic vs. placebo, or T1 vs. T2). This image-based strategy, when supported by prior marker selection and chemometric analysis, represents a complementary tool for exploring intra-individual trajectories and opens new perspectives for the application of fecal volatilomics in the context of personalized nutrition.

### Integrative clinical and microbiome findings

The companion clinical and metagenomic investigation performed on the same cohort of NCGWS subjects provides a complementary view of the biochemical and microbiological processes underlying the volatilomic patterns described here [49]. In this randomized, double-blind, placebo-controlled trial, probiotic supplementation (with *Lactiplantibacillus plantarum*, *Lacticaseibacillus paracasei*, and *Ligilactobacillus salivarius*) led to improved tolerance to gluten reintroduction in nearly half (47%) of the treated individuals, whereas no improvement was observed in the placebo group. Shotgun metagenomics revealed a significant increase in beneficial species such as *L. plantarum*, *Bifidobacterium adolescentis*, and *Coprococcus catus*, along with a reduction in pro-inflammatory taxa including *Bacteroides vulgatus* and *B. dorei*.

Functional analyses showed enrichment of genes related to bacteriocin biosynthesis, carbohydrate and protein metabolism, and particularly gliadin-degrading peptidases (e.g., glutaminase and proline iminopeptidase), supporting the hypothesis of enhanced gluten detoxification capacity. Gluten-tolerant participants displayed higher levels of propanoic acid and phenolic metabolites with known gut-protective and anti-inflammatory effects, which align with the volatilomic evidence of increased SCFA esters and reduced oxidative aldehydes. Together, these findings indicate that probiotics may mitigate gluten-related symptoms in NCGWS by reshaping the gut microbiota toward a metabolically efficient and less inflammatory configuration.

## Conclusions

The present study demonstrates that comprehensive GC×GC‐TOFMS significantly improves fecal volatilome profiling and expands fingerprinting accuracy and information potential, resolving more than 1000 recurring features (of which 270 were consistently identified) and providing three to four times higher peak capacity than one-dimensional GC-MS platforms used in previous studies on fecal volatilomics. This improved resolution allowed us to track subtle, diet-dependent changes in volatile organic compounds without the need for absolute quantification, thereby capturing metabolic shifts directly relevant to celiac disease and gluten-sensitive physiology. Moreover, the identification of 90 previously unreported compounds expanded the current knowledge of the fecal volatilome by approximately 20%, further underscoring the enhanced analytical depth and discovery potential achieved with the GC×GC‐TOFMS platform.

Our untargeted–targeted fingerprinting approach confirmed and extended the volatile key features first described by Ferrocino et al. [49] in the same clinical cohort. In particular, esters of short-chain fatty acids were repeatedly identified as the most responsive class of volatiles, increasing markedly under probiotic supplementation during both gluten withdrawal and gluten reintroduction. Probiotic-treated participants also exhibited a pronounced decrease in lipid-derived aldehydes and alcohols, suggesting a shift away from oxidative lipolysis products toward a more fermentation-efficient gut environment. By combining Fisher ratio filtering with dimension-reduction techniques, we were able to retain a minimal number of highly discriminatory features even after gluten reintroduction and still capture the probiotic-associated shifts that eluded previous linear models.

This comprehensive fingerprinting revealed a reproducible, non-invasive panel of VOC markers—elevated SCFA esters alongside reduced oxidative aldehydes and modulated indole-derived compounds—that faithfully reflects microbial adaptation to both dietary gluten and probiotic therapy. These volatilomic changes are consistent with metagenomic evidence of increased abundance of SCFA-producing and gliadin-degrading bacteria, reinforcing the link between microbial functionality and host metabolic response.

In this way, our work confirms the practical utility of high-resolution volatilomics for non-invasive monitoring of gut metabolic activity in celiac and gluten-sensitive populations. Larger cohort studies will be required in the future to validate these VOC biomarkers and determine their prognostic and diagnostic value. Deeper integration with metagenomic and transcriptomic analyses promises to elucidate the precise microbial pathways driving these volatilome changes, while longitudinal studies under different probiotic formulations and dietary regimens will help define the durability and specificity of probiotic-induced metabolic modulation. Ultimately, this integrated volatilomic framework could enable personalized nutritional and therapeutic strategies to improve gut health and gluten tolerance.

## Supplementary Information

Below is the link to the electronic supplementary material.

Supplementary Material 1 (PDF 130 KB)

Supplementary Material 2 (PDF 434 KB)

## Data Availability

Data has been uploaded to the Open Science Framework (OSF) website in a dedicated repository: https://osf.io/yk2g9/?view_only=6629ff4d1648441592239d58dc34a2cc. The access is made available upon request.
