# Supplementary material for: Advancing fecal volatilome profiling by comprehensive two-dimensional gas chromatography-time-of-flight mass spectrometry and image pattern recognition
Source: Anal Bioanal Chem. 2026 Jan 8;418(10):3037–58. doi: 10.1007/s00216-025-06280-6 (PMC13144212; doi:10.1007/s00216-025-06280-6)
Supplement: Supplementary file 1 — Supplementary Material 1 (PDF 130 KB) [file 216_2025_6280_MOESM1_ESM.pdf]

n.	Compound Name	Retention I (min) (mean)	%RSD	Retention II (sec) (mean)	%RSD	SNR (mean)	%RSD	LRI (experimental)	LRI (tabulated)	Delta (LRItab-LRIexp)	Chemical class
1	Ethanol	6.53	0.22	0.28	1.31	2220	2.18	906	920	14	Alcohols
2	Cyclohexanemethanol	24.33	0.22	0.59	1.62	277	1.31	1541	ND	ND	Alcohols
3	3-Methyl-3-buten-1-ol	14.53	0.22	0.43	7.85	1094	6.85	1234	1235	1	Alcohols
4	3-(Methylthio)-1-propanol	28.87	0.19	0.42	2.31	288	2.22	1703	1710	7	Alcohols
5	2-Undecanol	28.93	0.05	0.97	2.11	1227	3.99	1705	1706	1	Alcohols
6	2-Tridecanol	34.33	0.25	1.10	1.99	380	4.34	1898	1898	0	Alcohols
7	2-Propyl-1-pentanol	22.20	0.21	0.70	1.78	320	4.69	1464	ND	ND	Alcohols
8	2-Propanol	6.40	0.41	0.31	4.23	1618	4.19	898	915	17	Alcohols
9	2-Nonanol	23.07	0.43	0.83	5.19	1656	4.27	1495	1505	10	Alcohols
10	2-Methyl-2-buten-4-ol	16.80	0.30	0.42	4.98	302	5.38	1301	1311	10	Alcohols
11	2-Heptanol	16.73	0.21	0.67	2.11	765	2.64	1299	1298	-1	Alcohols
12	2-Furanmethanol	27.33	0.14	0.30	4.04	1288	6.24	1648	1635	-13	Alcohols
13	2-Decanol	25.13	0.56	0.89	3.88	203	5.67	1609	1621	12	Alcohols
14	2-Butanol	8.20	0.32	0.39	3.58	1507	5.24	1020	1019	-1	Alcohols
15	2-(2-Ethoxyethoxy)ethanol	26.20	0.41	0.50	1.47	380	5.08	1607	1615	8	Alcohols
16	2-(2-Butoxyethoxy)ethanol	31.13	0.22	0.61	5.19	270	8.68	1784	1796	12	Alcohols
17	2,3-Butanediol	24.73	0.23	0.31	1.54	424	1.77	1555	1542	-13	Alcohols
18	2-Methyl-1-butanol	13.13	0.06	0.48	1.78	5836	6.61	1192	1193	1	Alcohols
19	1-Tetradecanol	40.47	0.40	1.03	3.83	1167	1.78	2169	2173	4	Alcohols
20	2-Methyl-1-propanol	9.80	0.52	0.40	3.75	2453	3.37	1080	1085	5	Alcohols
21	1-Propanol	8.53	0.33	0.34	1.99	1052	2.32	1012	1030	18	Alcohols
22	1-Phenyl-2-propanol	31.87	0.03	0.53	1.13	202	1.54	1810	1778	-32	Alcohols
23	1-Pentanol	14.47	0.06	0.48	2.14	3644	2.50	1232	1250	18	Alcohols
24	1-Pentadecanol	42.80	0.18	1.08	2.84	4987	2.86	2276	2254	-22	Alcohols
25	1-Octen-3-ol	20.93	0.03	0.63	3.65	1429	1.55	1424	1430	6	Alcohols
26	1-Octanol	24.20	0.67	0.69	7.29	4411	2.29	1536	1534	-2	Alcohols
27	1-Nonanol	27.27	0.20	0.74	4.25	738	0.95	1645	1640	-5	Alcohols
28	1-Hexanol	17.80	0.29	0.55	2.86	6536	3.37	1331	1340	9	Alcohols
29	1-Hexadecanol	45.07	0.16	1.13	2.07	2943	7.11	2379	2365	-14	Alcohols
30	1-Heptanol	21.07	0.16	0.61	3.05	3337	9.19	1428	1440	12	Alcohols
31	1-Dodecanol	35.53	0.14	0.92	7.26	2209	2.30	1941	1930	-11	Alcohols
32	1-Decanol	30.13	0.21	0.80	4.83	1230	4.88	1748	1750	2	Alcohols
33	1-Butanol	11.27	0.18	0.41	2.87	3403	1.53	1146	1150	4	Alcohols
34	2-Octanol	19.93	0.12	0.75	7.84	1480	8.21	1394	1400	6	Alcohols
35	(E)-2-Nonenal	23.73	0.92	1.17	1.68	1019	4.84	1519	1532	13	Aldehydes
36	(E)-2-Heptenal	17.13	0.37	0.96	2.47	1302	4.85	1311	1310	-1	Aldehydes
37	Pentanal	7.40	0.52	0.62	1.88	5614	2.09	972	984	12	Aldehydes
38	Undecanal	25.80	0.23	1.50	2.59	290	5.76	1593	1606	13	Aldehydes
39	Tridecanal	31.80	0.09	1.60	2.13	1217	3.59	1807	1810	3	Aldehydes
40	Tetradecanal	34.60	0.45	1.65	1.98	4215	1.99	1908	1910	2	Aldehydes
41	2-Methylpropanal	5.20	0.74	0.39	3.99	4221	1.09	834	830	-4	Aldehydes
42	Propanal	5.00	0.42	0.32	2.07	337	4.67	814	800	-14	Aldehydes
43	Pentadecanal	37.20	0.42	1.70	2.55	3118	2.37	2020	2030	10	Aldehydes
44	Octanal	15.93	0.27	1.21	2.74	1493	1.62	1280	1295	15	Aldehydes
45	Octadecanal	44.53	0.30	1.79	3.67	205	9.41	2355	2350	-5	Aldehydes
46	Nonanal	19.33	0.64	1.33	2.55	3090	2.59	1397	1395	-2	Aldehydes
47	Methional	21.27	0.35	0.58	2.36	3622	1.53	1446	1454	8	Aldehydes
48	Hexanal	9.80	0.12	0.86	1.26	9858	5.31	1084	1088	4	Aldehydes
49	Hexadecanal	38.60	0.08	1.76	1.71	682	4.61	2084	2100	16	Aldehydes
50	Heptanal	12.73	0.73	1.05	6.56	2263	4.33	1180	1184	4	Aldehydes
51	Furfural	21.60	0.27	0.42	5.56	4026	1.99	1444	1450	6	Aldehydes
52	Dodecanal	28.87	0.21	1.56	2.35	4628	4.14	1703	1716	13	Aldehydes
53	Decanal	22.60	0.09	1.43	2.43	1028	5.18	1479	1480	1	Aldehydes
54	3-Methylbutanal	6.40	0.07	0.56	5.92	11367	5.83	918	916	-2	Aldehydes
55	Butanal	5.87	0.24	0.44	2.12	2016	1.52	870	877	7	Aldehydes
56	Benzeneacetaldehyde	27.00	0.13	0.60	6.19	9203	2.18	1636	1636	0	Aldehydes
57	Benzaldehyde	23.47	0.15	0.59	8.38	15278	3.37	1510	1508	-2	Aldehydes
58	Acetaldehyde	4.53	0.43	0.24	2.18	342	3.38	799	780	-19	Aldehydes
59	5-Methyl-2-furfural	24.93	0.33	0.54	13.93	2519	6.78	1562	1562	0	Aldehydes
60	4-Heptenal	14.47	0.00	0.88	4.76	204	2.13	1232	1243	11	Aldehydes
61	4-(1-Methylethyl)-benzaldehyde	30.93	0.27	0.83	10.96	997	3.01	1776	1770	-6	Aldehydes
62	3-Methyl-2-butenal	13.07	0.06	0.67	10.13	358	4.12	1190	1200	10	Aldehydes
63	2-Phenyl-2-butenal	34.73	0.09	0.70	3.38	563	4.79	1912	1922	10	Aldehydes
64	2-Hexenal	13.73	0.17	0.84	5.62	708	0.31	1210	1216	6	Aldehydes
65	2-Butenal	8.80	0.25	0.50	2.75	888	2.81	1042	1046	4	Aldehydes
66	(E,E)-2,4-Decadienal	31.60	0.07	0.98	2.07	1150	1.80	1800	1800	0	Aldehydes
67	(E,E)-2,4-Nonadienal	28.60	0.20	0.92	4.93	576	2.14	1693	1690	-3	Aldehydes
68	(Z)-9-Octadecenal	51.41	0.07	1.69	2.46	92	2.54	2685	2693	8	Aldehydes
69	(E,E)-2,4-Heptadienal	22.47	0.65	0.76	1.34	270	3.72	1474	1474	0	Aldehydes
70	p-Methylacetophenone	30.67	1.18	0.72	6.78	162	5.74	1767	1751	-16	Aromatic compounds
71	p-Methoxy-β-methylstyrene	32.07	0.00	0.76	2.06	11889	7.98	1817	1820	3	Aromatic compounds
72	p-Cresol	41.00	0.21	0.27	3.10	564	2.75	2194	2180	-14	Aromatic compounds
73	o-Cymene	15.40	1.04	1.21	5.76	4116	3.24	1259	1268	9	Aromatic compounds

Chemical Class	N. of features
Alcohols	34
Aldehydes	35
Aromatic compounds	20
Carboxylic acids	18
Esters	24
Heterocyclic compounds	15
Hydrocarbons	12
Isothiocyanates	2
Ketones	40
Lactones	4
Nitrogen Compounds	8
Pyrazines	3
Sulfides	5
Terpenes	50
Tot TARGETED	270
Tot UT	1037

74	Styrene	14.93	0.17	0.74	9.07	2869	3.54	1246	1260	14	Aromatic compounds
75	Phenol	36.53	0.76	0.29	15.38	4607	2.94	1990	1987	-3	Aromatic compounds
76	Phenethyl acetate	31.87	0.11	0.73	5.34	1255	6.56	1810	1820	10	Aromatic compounds
77	Methyl salicylate	30.87	0.62	0.66	4.19	397	4.55	1762	1747	-15	Aromatic compounds
78	Ethylbenzene	11.40	0.26	0.89	3.08	238	1.45	1135	1125	-10	Aromatic compounds
79	Butyl Hydroxytoluene	34.33	0.95	1.19	0.04	237	1.13	1898	1898	0	Aromatic compounds
80	4-Methoxytoluene	20.73	0.20	0.77	1.91	388	5.19	1418	1430	12	Aromatic compounds
81	4-Isopropyl-1,6-dimethylnaphthalene	41.80	0.03	1.02	7.46	887	5.92	2230	2220	-10	Aromatic compounds
82	4-Ethylphenol	40.53	0.03	0.35	0.53	1228	8.25	2172	2170	-2	Aromatic compounds
83	3-Methylphenol	38.33	0.66	0.33	3.22	8393	0.13	2072	2081	9	Aromatic compounds
84	2-Ethyltoluene	15.73	0.20	1.05	1.44	155	1.64	1269	1260	-9	Aromatic compounds
85	2,4-Di-tert-butylphenol	43.60	0.07	0.57	2.81	867	4.94	2312	2315	3	Aromatic compounds
86	1-Ethenyl-2-methyl benzene	18.20	0.23	0.85	6.79	379	1.66	1343	1342	-1	Aromatic compounds
87	(E)-1-Butenylbenzene	21.07	0.60	0.96	6.42	355	4.96	1468	1479	11	Aromatic compounds
88	1-Methyl-4-isopropenylbenzene	20.67	0.34	0.96	3.57	1278	1.78	1416	1420	4	Aromatic compounds
89	1,1,6-Trimethyl-1,2-dihydro naphthalene	34.53	0.24	1.30	2.81	635	7.59	1765	1751	-14	Aromatic compounds
90	Pentanoic acid	30.07	0.23	0.28	7.32	6628	3.67	1746	1744	-2	Carboxylic acids
91	Acetic acid	21.80	0.19	0.19	4.85	2333	1.43	1450	1450	0	Carboxylic acids
92	Tridecanoic acid	52.60	1.03	0.48	6.42	404	3.34	2713	2703	-10	Carboxylic acids
93	2-Methylpropanoic acid	25.40	0.55	0.25	3.92	2960	3.90	1579	1587	8	Carboxylic acids
94	Propanoic acid	24.33	0.32	0.23	3.26	3940	2.13	1541	1530	-11	Carboxylic acids
95	Octanoic acid	38.67	0.12	0.34	1.50	2487	1.42	2083	2070	-13	Carboxylic acids
96	Nonanoic acid	41.40	0.11	0.35	4.66	2678	4.14	2212	2211	-1	Carboxylic acids
97	Hydrocinnamic acid	52.27	0.29	0.25	5.65	269	4.02	2648	2638	-10	Carboxylic acids
98	Hexanoic acid	32.93	0.43	0.31	4.91	7474	8.34	1848	1850	2	Carboxylic acids
99	Heptanoic acid	35.87	0.19	0.32	7.04	7238	6.00	1953	1950	-3	Carboxylic acids
100	Dodecanoic acid	48.33	0.13	0.43	4.77	2764	1.98	2528	2523	-5	Carboxylic acids
101	Decanoic acid	43.80	0.20	0.38	1.12	4332	4.05	2291	2280	-11	Carboxylic acids
102	2-Methylbutanoic acid	28.20	0.05	0.28	1.99	7170	1.48	1679	1670	-9	Carboxylic acids
103	Butanoic acid	26.87	0.26	0.26	3.54	8757	5.56	1631	1622	-9	Carboxylic acids
104	5-Methylhexanoic acid	35.07	0.37	0.30	1.67	711	8.18	1924	1914	-10	Carboxylic acids
105	(E)-6,10-Dimethyl-5,9-undecadien-2-one	32.80	0.18	1.23	0.71	783	5.40	1843	1864	21	Carboxylic acids
106	4-Methyl-2-pentanone	8.00	0.41	0.76	3.81	1873	2.06	984	1010	26	Carboxylic acids
107	4-Methyl-pentanoic acid	32.33	1.12	0.28	7.53	601	4.55	1827	1817	-10	Carboxylic acids
108	Methyl butanoate	7.60	0.60	0.63	4.34	700	5.39	989	989	0	Esters
109	Ethyl hexadecanoate	42.53	0.33	1.90	5.77	244	5.51	2264	2250	-14	Esters
110	Butyl butanoate	15.63	0.33	1.41	2.41	9013	3.07	1234	1245	11	Esters
111	Ethyl pentanoate	12.73	0.21	1.14	3.10	4321	1.92	1146	1139	-7	Esters
112	Ethyl butanoate	9.82	0.75	0.86	4.13	3251	2.38	1037	1044	7	Esters
113	Ethyl propanoate	8.01	0.65	0.61	6.00	1975	4.11	957	966	9	Esters
114	Ethyl hexanoate	16.17	0.39	1.33	2.99	1788	1.25	1246	1246	0	Esters
115	Propyl butanoate	12.39	0.32	1.14	3.26	1428	3.63	1144	1153	9	Esters
116	Ethyl octanoate	24.79	0.13	2.13	1.67	1379	2.84	1466	1463	-3	Esters
117	Butyl isobutanoate	13.17	0.42	1.43	2.55	1361	4.53	1158	1149	-9	Esters
118	Ethyl isobutanoate	8.42	0.52	0.73	4.61	1215	8.74	982	971	-11	Esters
119	Methyl trimethylacetate	7.34	0.32	0.66	5.31	1130	6.12	911	887	-24	Esters
120	Ethyl 3-methylbutanoate	10.68	0.41	1.06	3.32	995	2.47	1069	1056	-13	Esters
121	Ethyl 2-methylbutanoate	10.24	0.43	1.08	3.41	570	3.05	1054	1062	8	Esters
122	Decyl decanoate	55.67	0.00	0.70	4.76	439	0.88	2561	2565	4	Esters
123	Propyl 3-methylbutanoate	13.39	0.45	1.38	2.89	389	2.33	1164	1145	-19	Esters
124	Methyl isovalerate	9.38	0.40	0.78	4.55	274	4.18	1022	1022	0	Esters
125	n-Pentyl butanoate	17.40	0.34	1.57	3.57	247	1.65	1280	1305	25	Esters
126	Ethyl 2-phenylacetate	35.29	0.20	0.77	4.93	224	5.12	1771	1768	-3	Esters
127	3-Methyl-1-butyl butanoate	16.72	0.27	1.60	2.29	222	6.47	1258	1255	-3	Esters
128	Butyl 2-methylbutanoate	16.14	0.15	1.67	2.44	205	7.03	1245	1219	-26	Esters
129	Ethyl 4-methylpentanoate	14.69	0.42	1.35	3.39	166	1.36	1204	1204	0	Esters
130	Ethyl 3-phenylpropanoate	38.32	0.21	0.84	5.75	126	4.07	1872	1872	0	Esters
131	Butyl benzoate	33.13	0.27	0.93	2.22	1484	0.97	1855	1871	16	Esters
132	Pyrrole	23.20	0.05	0.30	7.20	2716	3.39	1500	1500	0	Heterocyclic compounds
133	Pyridine	12.40	1.24	0.54	7.61	1321	3.75	1169	1183	14	Heterocyclic compounds
134	Methylethylmaleimide	42.80	0.52	0.37	4.61	993	1.61	2255	2260	5	Heterocyclic compounds
135	Indole	46.40	0.32	0.38	5.31	12868	3.10	2440	2441	1	Heterocyclic compounds
136	5-Methylindole	47.40	0.31	0.43	3.96	13900	3.49	2486	2468	-18	Heterocyclic compounds
137	4-Methyl-5-(2-hydroxyethyl)thiazole	43.33	0.29	0.41	3.49	481	2.36	2300	2311	11	Heterocyclic compounds
138	3-Phenylfuran	32.80	0.22	0.61	1.71	193	1.88	1843	1839	-4	Heterocyclic compounds
139	3-Methylfuran	6.07	0.20	0.41	2.49	453	4.57	881	853	-28	Heterocyclic compounds
140	3,4-Dimethylthiophene	14.80	0.45	0.80	2.89	187	7.20	1242	1253	11	Heterocyclic compounds
141	2-Pentylfuran	14.13	0.18	1.13	2.17	3816	3.84	1222	1228	6	Heterocyclic compounds
142	2-Pentylthiophene	21.33	0.07	1.21	4.84	441	3.00	1436	1438	2	Heterocyclic compounds
143	2-Methylfuran	5.80	0.25	0.39	1.63	462	2.40	866	871	5	Heterocyclic compounds
144	2-Indolinone	54.07	0.27	0.39	2.65	676	4.85	2790	ND	ND	Heterocyclic compounds
145	2-Acetylfuran	22.87	0.29	0.51	10.95	2180	2.43	1488	1488	0	Heterocyclic compounds
146	2-Acetyl-5-methylfuran	26.13	0.08	0.61	3.06	387	1.94	1605	1603	-2	Heterocyclic compounds
147	Tridecane	16.20	0.28	3.57	4.33	404	4.19	1300	1300	0	Hydrocarbons
148	Tetradecane	19.40	0.29	3.65	7.23	2700	5.94	1400	1400	0	Hydrocarbons
149	Pentadecane	22.53	0.46	3.66	3.90	2353	4.06	1500	1500	0	Hydrocarbons

150	Octadecane	31.33	0.32	3.53	8.34	3070	2.99	1798	1800	2	Hydrocarbons
151	Hexadecane	25.60	0.17	3.62	6.24	3136	2.55	1600	1600	0	Hydrocarbons
152	Heptane	4.53	0.41	0.55	3.32	209	6.30	700	700	0	Hydrocarbons
153	Heptadecane	28.53	0.26	3.58	3.34	1712	8.04	1700	1700	0	Hydrocarbons
154	Dodecane	13.00	1.00	3.38	8.66	1241	2.95	1200	1200	0	Hydrocarbons
155	Decane	7.67	0.03	2.26	6.94	214	4.83	1000	1000	0	Hydrocarbons
156	Cyclopentanone	12.73	0.24	0.72	11.99	235	2.39	1180	1180	0	Hydrocarbons
157	Cyclohexane	4.67	0.27	0.49	2.70	254	1.12	722	732	10	Hydrocarbons
158	Octane	5.07	0.14	0.90	2.62	612	7.95	800	800	0	Hydrocarbons
159	Allyl Isothiocyanate	18.20	0.07	0.61	0.95	4676	5.62	1343	1356	13	Isothiocyanates
160	3-Butenyl isothiocyanate	21.27	0.11	0.73	4.54	1205	1.18	1434	1459	25	Isothiocyanates
161	Phenylacetone	29.40	0.51	0.68	3.84	1435	3.38	1722	1710	-12	Ketones
162	(E)-2-Octenal	20.47	0.12	1.07	14.02	1602	2.60	1415	1430	15	Ketones
163	Isopropenyl ethyl ketone	9.40	0.44	0.74	4.79	1702	8.66	1059	1069	10	Ketones
164	Acetophenone	27.20	0.49	0.64	6.60	1605	2.41	1643	1623	-20	Ketones
165	Acetone	5.20	0.31	0.33	1.64	6386	3.85	822	814	-8	Ketones
166	Allyl methyl disulfide	15.67	0.07	0.81	4.58	222	8.89	1267	1266	-1	Ketones
167	6-Methyl-5-hepten-2-one	17.53	0.09	0.97	7.98	4173	3.95	1323	1323	0	Ketones
168	(E)-6-Methyl-3,5-heptadien-2-one	25.40	0.09	0.83	2.85	165	3.57	1579	1582	3	Ketones
169	6-Methyl-2-heptanone	14.33	0.26	1.18	7.71	321	2.59	1228	1228	0	Ketones
170	5-Methyl-3-hexanone	9.60	0.31	1.04	3.55	432	2.74	1069	1060	-9	Ketones
171	5-Methyl-2-hexanone	11.40	0.20	1.01	7.69	232	11.55	1135	1150	15	Ketones
172	5-Methyl-2-heptanone	14.87	0.57	1.18	1.94	295	5.46	1244	1252	8	Ketones
173	4-Cyclopentene-1,3-dione	25.40	0.34	0.44	5.50	392	3.50	1579	1605	26	Ketones
174	3-Undecen-2-one	29.20	0.35	1.27	11.56	365	1.82	1715	1710	-5	Ketones
175	3-Penten-2-one	10.93	0.15	0.63	5.77	2204	2.62	1119	1121	2	Ketones
176	3-Octen-2-one	19.73	0.39	1.05	1.19	1313	4.98	1388	1410	22	Ketones
177	3-Nonen-2-one	23.00	0.09	1.13	1.80	235	9.69	1493	1515	22	Ketones
178	3-Methyl-2-pentanone	8.20	0.28	0.80	3.90	649	3.11	995	1016	21	Ketones
179	3-Methyl-2-butanone	6.60	0.12	0.58	1.90	811	0.06	909	929	20	Ketones
180	3-Hexanone	9.00	0.20	0.86	4.85	792	4.00	1037	1040	3	Ketones
181	3,5,5-trimethyl-2-Cyclohexen-1-one	25.53	0.19	1.00	3.07	213	3.08	1583	1576	-7	Ketones
182	2-Undecanone	25.60	0.86	1.47	2.49	1188	3.92	1586	1580	-6	Ketones
183	2-Tridecanone	31.60	0.19	1.58	4.14	1048	5.34	1800	1814	14	Ketones
184	2-Pentadecanone	37.00	0.27	1.67	3.92	1708	4.92	2011	2021	10	Ketones
185	2-Octanone	15.80	0.05	1.20	1.97	2322	5.13	1271	1287	16	Ketones
186	2-Nonanone	19.13	0.36	1.31	8.68	1697	4.91	1371	1370	-1	Ketones
187	2-Hydroxy-3-pentanone	18.33	0.08	0.49	2.30	311	2.85	1347	1360	13	Ketones
188	2-Heptanone	12.60	0.15	1.04	2.24	3816	7.29	1175	1173	-2	Ketones
189	2-Dodecanone	28.67	0.45	1.53	5.18	327	1.98	1695	1704	9	Ketones
190	2-Decanone	22.47	0.29	1.40	2.40	413	2.23	1474	1480	6	Ketones
191	3-Hydroxy-2-butanone	16.00	0.18	0.41	2.67	456	4.77	1277	1271	-6	Ketones
192	2-Butanone	6.13	0.50	0.46	4.18	5035	1.67	904	905	1	Ketones
193	2,3-Hexanedione	11.07	0.19	0.69	4.04	338	5.19	1123	1136	13	Ketones
194	2,3-Heptanedione	11.73	0.40	0.82	4.55	761	1.94	1146	1153	7	Ketones
195	2,3-Butanedione	7.47	0.36	0.40	10.53	4021	4.60	955	978	23	Ketones
196	1-Octen-3-one	16.33	0.33	1.04	5.74	167	6.93	1287	1298	11	Ketones
197	1-Acetylcyclohexene	11.00	0.19	0.95	4.19	309	3.43	1121	ND	ND	Ketones
198	3-Octanone	14.87	0.15	1.26	3.20	246	3.11	1244	1244	0	Ketones
199	2-Tetradecanone	34.33	0.08	1.63	2.62	158	5.47	1898	1900	2	Ketones
200	2,3-Pentanedione	9.20	0.27	0.55	2.29	4341	5.73	1048	1056	8	Ketones
201	δ-Caprolactone	31.13	0.06	0.68	11.55	749	6.14	1784	1780	-4	Lactones
202	γ-Dodecalactone	45.13	0.22	1.01	3.67	1768	2.35	2382	2381	-1	Lactones
203	γ-Hexalactone	32.58	0.15	0.69	5.19	191	1.44	1692	1694	2	Lactones
204	Dihydro-5-pentyl-2(3H)-Furanone	37.27	0.11	0.87	4.06	301	4.36	2023	2023	0	Lactones
205	Trimethylamine	4.13	0.31	0.25	6.00	431	3.56	622	609	-13	Nitrogen compounds
206	Hexanamide	48.20	0.13	0.55	4.05	685	5.76	2522	2520	-2	Nitrogen compounds
207	Benzonitrile	26.00	0.26	0.56	7.00	300	3.92	1600	1583	-17	Nitrogen compounds
208	Acetonitrile	8.00	0.16	0.31	2.75	231	2.44	984	1012	28	Nitrogen compounds
209	Acetamide	29.73	0.51	0.26	3.14	445	2.46	1734	1748	14	Nitrogen compounds
210	2-Furancarbonitrile	19.33	0.00	0.45	1.80	312	1.75	1377	ND	ND	Nitrogen compounds
211	2-Acetylaniline	41.60	0.17	0.47	2.83	707	2.79	2196	2202	6	Nitrogen compounds
212	1-Vinylaziridine	7.80	0.11	0.56	1.18	1097	1.93	973	ND	ND	Nitrogen compounds
213	Trimethylpyrazine	19.47	0.05	0.79	3.00	165	4.93	1381	1389	8	Pyrazines
214	Tetramethylpyrazine	21.67	0.62	0.88	1.82	361	2.84	1446	1460	14	Pyrazines
215	2,5-Dimethylpyrazine	17.07	0.11	0.69	5.63	426	1.71	1309	1320	11	Pyrazines
216	Methylpropyl disulfide	14.13	0.07	0.98	1.45	161	1.62	1222	1230	8	Sulfides
217	Dimethyl trisulfide	18.87	1.58	0.79	2.95	3426	7.71	1363	1362	-1	Sulfides
218	Dimethyl sulfide	4.73	0.21	0.32	2.60	1109	1.90	789	773	-16	Sulfides
219	Dimethyl disulfide	9.53	0.44	0.63	4.12	8730	2.11	1066	1065	-1	Sulfides
220	Allyl methyl sulfide	8.13	0.13	0.63	2.66	725	2.47	981	970	-11	Sulfides
221	(Z)-β-Ocimene	15.13	0.29	1.39	5.94	1916	1.34	1251	1240	-11	Terpenes
222	γ-Terpinene	14.53	0.00	1.47	9.41	5839	4.23	1234	1250	16	Terpenes
223	γ-Elemene	26.87	0.33	1.81	2.59	574	4.69	1631	1633	2	Terpenes
224	β-Terpinyl acetate	28.60	0.08	1.24	3.30	940	3.67	1633	1622	-11	Terpenes
225	β-Phellandrene	13.40	0.01	1.52	4.38	2564	6.80	1200	1200	0	Terpenes

226	β-Myrcene	12.00	0.36	1.37	7.52	1499	5.65	1155	1150	-5	Terpenes
227	β-Farnesene	27.53	0.33	1.75	2.41	644	7.23	1655	1660	5	Terpenes
228	β-Elemene	25.47	0.05	1.88	5.76	2160	2.43	1581	1580	-1	Terpenes
229	β-Bisabolene	29.33	0.06	1.76	8.25	5114	7.32	1719	1715	-4	Terpenes
230	α-Terpinene	12.53	0.06	1.52	3.72	374	5.77	1173	1170	-3	Terpenes
231	α-Pinene	8.33	0.26	1.53	6.25	3485	2.19	1002	1015	13	Terpenes
232	α-Phellandrene	12.07	0.27	1.50	13.01	1458	5.94	1157	1160	3	Terpenes
233	α-Curcumene	30.67	0.37	1.45	10.89	1764	4.76	1767	1770	3	Terpenes
234	α-Copaene	22.20	0.10	2.47	3.50	3241	7.22	1464	1480	16	Terpenes
235	α-Caryophyllene	27.87	0.37	1.93	8.69	5024	1.56	1667	1665	-2	Terpenes
236	α-Bergamotene	24.80	1.05	2.10	3.26	360	8.50	1557	1570	13	Terpenes
237	(E)-Calamenene	32.33	0.25	1.49	14.11	3718	7.00	1827	1820	-7	Terpenes
238	(E)-Bergamotene	25.33	0.34	2.14	6.30	6688	6.42	1576	1570	-6	Terpenes
239	(E)-3-Hepten-2-one	16.33	0.23	0.94	12.43	120	3.96	1287	1274	-13	Terpenes
240	δ-Elemene	21.80	0.15	2.23	9.69	2151	3.67	1450	1460	10	Terpenes
241	α-Thujene	8.40	0.17	1.44	6.80	1837	1.26	1005	1015	10	Terpenes
242	α-Cubebene	21.40	0.75	2.44	4.13	3946	1.87	1438	1440	2	Terpenes
243	Terpinolene	15.73	0.70	1.47	18.18	1108	6.25	1269	1280	11	Terpenes
244	Sesquiterpenoid 9	27.20	0.00	1.95	9.19	743	10.26	1643	ND	ND	Terpenes
245	Sesquiterpenoid 8	26.20	0.37	2.06	2.94	1064	5.68	1607	ND	ND	Terpenes
246	Sesquiterpenoid 7	25.93	0.14	2.10	2.95	1088	3.41	1598	ND	ND	Terpenes
247	Sesquiterpenoid 6	25.07	0.65	2.13	6.00	1732	6.00	1567	ND	ND	Terpenes
248	Sesquiterpenoid 5	25.47	0.32	2.18	3.95	633	2.29	1581	ND	ND	Terpenes
249	Sesquiterpenoid 4	24.33	0.41	2.22	2.09	158	3.32	1541	ND	ND	Terpenes
250	Sesquiterpenoid 3	23.93	0.48	2.24	6.59	529	2.89	1526	ND	ND	Terpenes
251	Sesquiterpenoid 2	23.47	0.45	2.29	14.00	523	4.13	1510	ND	ND	Terpenes
252	Sesquiterpenoid 17	31.20	0.12	1.66	4.14	289	3.26	1786	ND	ND	Terpenes
253	Sesquiterpenoid 16	30.93	0.58	1.69	8.87	958	3.30	1776	ND	ND	Terpenes
254	Sesquiterpenoid 15	30.93	0.39	1.57	2.99	564	1.03	1776	ND	ND	Terpenes
255	Sesquiterpenoid 14	30.67	0.46	1.67	4.57	480	7.52	1767	ND	ND	Terpenes
256	Sesquiterpenoid 13	28.93	0.15	1.89	2.37	1969	2.59	1705	ND	ND	Terpenes
257	Sesquiterpenoid 12	28.40	0.17	1.88	2.13	5879	1.80	1686	ND	ND	Terpenes
258	Sesquiterpenoid 11	27.53	0.52	1.99	8.50	313	4.76	1655	ND	ND	Terpenes
259	Sesquiterpenoid 10	27.40	0.17	1.95	4.36	613	4.38	1650	ND	ND	Terpenes
260	Sesquiterpenoid 1	30.20	0.47	1.74	5.46	3068	1.19	1750	ND	ND	Terpenes
261	Sabinene	10.80	0.19	1.51	2.47	3036	4.11	1114	1115	1	Terpenes
262	Perillene	20.20	0.13	1.02	1.67	191	1.99	1415	1430	15	Terpenes
263	Menthol	26.80	0.80	0.84	4.69	2433	2.47	1629	1630	1	Terpenes
264	Linalool	24.00	1.00	0.76	5.13	1156	1.71	1529	1530	1	Terpenes
265	Limonene	13.07	0.07	1.51	1.22	20095	6.86	1190	1210	20	Terpenes
266	3-Isopropyl-6-methyl-1-cyclohexene	11.60	0.37	1.71	3.48	5197	2.17	1141	ND	ND	Terpenes
267	δ-Carene	11.60	0.16	1.59	3.11	4424	0.95	1141	1149	8	Terpenes
268	Aromadendrene	26.47	0.43	2.09	3.41	351	1.29	1617	1620	3	Terpenes
269	3-Isopropyl-6-methylcyclohexene	12.20	0.67	2.02	5.51	219	9.22	1162	1160	-2	Terpenes
270	2-Methyl-5-isopropenyl-2-cyclohexenone	29.60	1.39	0.94	5.83	2583	17.46	1729	1725	-4	Terpenes
271	#7	53.78	0.29	0.41	1.44	8870	2.99	2489			
272	#21	6.20	0.11	0.97	18.77	7324	3.71	843			
273	#22	7.41	0.14	0.26	5.07	7723	1.14	925			
274	#29	8.27	0.11	2.29	1.75	4660	19.91	971			
275	#41	30.14	0.10	0.49	7.43	411	10.49	1632			
276	#42	12.72	0.01	3.35	2.07	2521	5.35	1146			
277	#44	26.57	0.06	1.29	15.56	233	3.61	1517			
278	#45	4.53	0.43	0.92	1.52	338	9.07	589			
279	#51	5.93	0.27	0.35	2.51	3434	1.50	822			
280	#59	8.20	0.38	0.76	2.29	1753	6.79	968			
281	#60	20.01	0.16	0.72	4.32	104	8.87	1345			
282	#61	23.32	0.26	1.09	2.25	681	2.29	1429			
283	#65	11.21	0.23	0.86	4.15	762	7.53	1089			
284	#68	4.55	0.51	0.42	2.62	169	1.00	589			
285	#70	31.61	0.06	0.18	12.55	742	2.49	1671			
286	#71	30.52	0.21	0.81	7.41	1877	2.96	1648			
287	#73	31.80	0.36	0.45	9.64	265	2.10	1674			
288	#74	19.19	0.31	1.57	10.49	647	4.11	1325			
289	#75	33.46	0.07	1.89	7.08	829	4.05	1719			
290	#79	43.54	0.06	0.66	2.23	1537	2.24	2057			
291	#80	6.88	0.18	0.88	8.87	1392	2.11	895			
292	#84	7.02	0.17	0.87	9.19	1370	9.18	904			
293	#85	7.24	0.15	0.85	2.44	1451	7.20	918			
294	#87	7.37	0.18	0.84	1.68	1401	1.66	925			
295	#88	30.89	0.67	0.66	13.25	1385	2.75	1653			
296	#90	7.97	0.12	0.77	3.37	1187	3.10	954			
297	#91	8.60	0.12	0.71	4.67	1184	5.71	986			
298	#92	7.63	0.19	0.80	6.86	1202	3.34	936			
299	#101	8.84	0.23	0.69	1.14	1131	3.34	1000			
300	#102	58.67	0.17	1.40	6.08	2946	8.01	2678			
301	#104	8.99	0.40	0.68	2.46	1132	1.47	1007			

302	#106	9.35	0.46	0.65	1.47	1153	3.04	1027
303	#107	9.17	0.17	0.67	2.95	1110	1.96	1012
304	#111	32.16	0.25	1.01	2.39	106	2.52	1683
305	#112	13.58	0.09	1.42	14.84	303	4.80	1173
306	#120	43.54	0.38	1.15	1.87	444	7.26	2057
307	#121	45.52	0.34	0.26	5.86	1387	5.92	2118
308	#124	34.22	0.33	0.46	3.37	200	6.08	1736
309	#127	30.07	0.50	1.19	2.01	508	2.07	1630
310	#129	30.59	0.18	0.40	4.63	393	5.19	1648
311	#135	24.23	0.29	0.75	9.18	252	2.62	1445
312	#136	34.14	0.15	0.68	5.19	211	1.96	1729
313	#138	27.61	0.06	0.66	3.56	1035	4.64	1546
314	#140	34.50	0.47	0.56	4.59	263	4.85	1745
315	#141	8.67	0.11	2.14	3.72	535	7.41	993
316	#143	30.44	0.30	1.33	12.24	158	3.20	1648
317	#146	32.07	0.21	0.47	4.02	208	6.62	1683
318	#147	28.92	0.32	0.61	6.58	179	1.87	1591
319	#149	16.04	0.26	1.20	2.52	233	6.48	1243
320	#151	27.27	0.60	0.76	2.79	813	2.36	1536
321	#152	8.81	0.16	2.10	5.67	483	3.14	1000
322	#155	34.47	0.04	0.67	3.92	151	4.59	1745
323	#159	58.68	0.22	1.91	2.52	742	2.24	2678
324	#163	19.95	0.22	1.33	5.46	272	4.18	1345
325	#164	29.39	0.18	1.08	2.35	109	4.81	1605
326	#166	6.66	0.12	0.39	4.39	352	4.00	879
327	#167	9.01	0.29	2.04	3.04	427	4.06	1007
328	#171	24.18	0.22	0.99	3.67	124	3.55	1445
329	#174	9.47	0.15	1.90	4.81	418	3.00	1025
330	#175	32.62	0.02	0.74	0.97	290	2.17	1696
331	#177	9.09	0.18	2.01	2.41	486	1.16	1010
332	#179	4.71	0.96	0.91	10.06	63	2.08	611
333	#180	30.79	0.14	0.38	7.21	221	2.81	1651
334	#182	34.48	0.42	0.95	3.39	128	1.54	1745
335	#184	24.24	0.44	0.91	3.71	104	3.07	1451
336	#185	27.24	0.22	0.54	1.52	139	2.50	1533
337	#186	30.16	0.37	1.68	3.24	30	3.32	1632
338	#188	9.34	0.61	1.93	4.88	375	1.13	1020
339	#192	34.27	0.16	1.72	5.39	149	2.57	1745
340	#193	30.16	0.35	1.75	1.85	28	1.47	1632
341	#194	34.02	0.05	0.97	7.22	65	1.84	1731
342	#199	30.17	0.25	1.81	5.71	27	2.53	1632
343	#201	16.43	0.11	1.36	4.53	246	1.79	1253
344	#204	31.49	0.11	0.65	2.36	56	2.87	1666
345	#205	34.54	0.23	0.33	5.01	211	1.14	1747
346	#206	30.57	0.77	1.88	18.98	60	10.71	1649
347	#209	22.14	0.10	3.83	4.13	103	1.68	1399
348	#210	32.41	0.18	0.32	5.24	266	2.95	1691
349	#211	24.07	0.36	1.16	5.87	199	2.00	1446
350	#212	37.12	0.50	0.76	3.37	53	1.81	1833
351	#213	25.05	0.80	0.57	9.91	206	2.07	1474
352	#215	29.26	0.37	1.51	1.98	337	13.25	1603
353	#216	9.17	0.22	0.78	1.27	162	6.19	1012
354	#217	30.93	0.20	0.30	1.96	274	1.85	1653
355	#218	15.61	1.40	1.10	13.31	40	2.63	1232
356	#219	34.20	0.27	1.37	3.90	50	1.63	1747
357	#220	32.33	0.23	0.87	4.05	96	5.01	1686
358	#221	30.65	0.48	1.99	2.24	58	2.39	1649
359	#222	27.30	1.16	0.60	3.33	78	2.88	1536
360	#223	32.47	0.25	0.59	3.91	81	1.96	1691
361	#224	52.67	0.08	0.66	13.17	747	2.39	2445
362	#225	36.07	0.31	1.62	6.61	258	13.31	1798
363	#227	29.74	0.36	0.60	10.09	90	9.95	1618
364	#229	12.75	0.09	1.35	7.13	80	7.13	1148
365	#230	16.53	0.35	0.90	1.16	164	4.83	1253
366	#231	9.61	0.20	3.04	3.87	202	3.42	1030
367	#232	47.28	0.71	0.22	9.40	148	1.99	2190
368	#233	27.73	0.00	0.53	5.94	209	3.83	1553
369	#236	27.19	0.37	0.67	5.78	72	7.04	1535
370	#237	9.80	0.23	1.11	5.11	69	3.46	1037
371	#238	18.06	0.10	0.38	2.12	2150	2.29	1295
372	#239	29.44	0.26	2.29	7.15	1346	5.12	1609
373	#240	30.55	0.29	1.29	4.76	68	19.31	1648
374	#243	19.00	0.22	0.39	4.39	207	7.98	1320
375	#244	31.87	0.24	1.50	7.98	59	3.23	1678
376	#245	4.95	0.16	0.44	3.23	458	1.98	656
377	#246	29.27	0.09	3.76	11.40	76	27.84	1603

378	#247	10.54	0.16	1.62	8.01	381	2.83	1067
379	#249	24.24	0.96	1.29	5.63	78	2.22	1453
380	#250	28.85	0.72	0.43	14.66	73	5.41	1596
381	#251	34.24	0.16	1.57	2.04	40	7.15	1745
382	#252	24.49	0.09	0.88	1.90	116	3.58	1459
383	#253	31.87	0.21	1.61	5.75	27	3.77	1678
384	#256	22.78	0.03	1.70	3.55	320	2.67	1416
385	#258	13.32	0.16	0.38	14.22	195	2.24	1164
386	#263	31.99	0.40	1.55	2.71	87	2.93	1683
387	#264	37.65	0.72	0.30	4.94	1251	2.94	1850
388	#266	17.02	0.10	1.63	8.83	53	4.85	1267
389	#267	33.95	0.22	1.80	3.00	23	4.04	1729
390	#270	12.41	0.09	0.62	12.76	161	5.09	1134
391	#272	34.27	0.07	1.88	3.61	37	2.21	1747
392	#273	23.97	0.16	1.44	15.49	49	3.37	1443
393	#275	16.47	0.05	0.39	4.38	130	5.07	1253
394	#276	10.98	0.21	3.36	2.99	201	6.38	1082
395	#277	34.79	0.68	0.35	2.57	174	5.31	1754
396	#278	27.66	0.17	0.54	1.96	433	1.97	1551
397	#279	11.34	0.11	1.43	6.69	178	1.68	1094
398	#280	45.22	0.23	1.05	11.53	38	39.62	2118
399	#283	35.43	0.21	0.65	3.09	239	4.32	1776
400	#284	34.53	0.15	1.35	4.05	86	2.75	1745
401	#285	31.23	0.11	0.57	5.56	202	4.58	1661
402	#286	42.27	0.31	1.71	8.95	216	17.94	2011
403	#287	28.92	0.15	0.69	1.18	62	4.83	1591
404	#289	11.21	0.14	1.46	3.31	212	1.75	1092
405	#290	31.84	0.69	1.75	19.13	20	5.19	1674
406	#292	34.35	0.07	2.02	0.07	32	37.12	1747
407	#293	27.29	0.49	0.86	17.94	103	2.41	1540
408	#294	28.22	0.08	0.53	2.99	211	2.62	1569
409	#297	27.09	1.04	1.17	13.03	229	2.46	1531
410	#298	28.95	0.50	1.00	1.72	75	4.39	1594
411	#299	9.75	0.08	1.82	4.91	200	23.99	1035
412	#300	35.33	0.14	0.66	4.26	94	5.77	1774
413	#301	25.81	0.27	1.46	5.08	159	2.25	1495
414	#302	27.41	0.19	1.18	1.33	531	2.24	1558
415	#303	14.05	0.18	1.06	4.83	190	8.18	1189
416	#304	31.93	0.14	1.78	3.24	27	12.76	1681
417	#305	24.61	0.15	0.78	1.20	57	1.90	1462
418	#306	19.39	0.38	0.71	1.85	171	2.35	1331
419	#307	24.26	0.39	1.50	4.27	43	10.09	1453
420	#308	9.91	0.37	1.07	1.97	47	4.13	1042
421	#309	17.16	0.27	1.00	3.66	495	2.37	1272
422	#310	30.78	0.39	1.23	6.85	123	3.84	1651
423	#313	32.11	0.18	1.87	4.96	48	17.46	1683
424	#315	9.74	0.15	3.07	0.95	139	3.72	1035
425	#317	33.97	0.24	2.00	1.23	20	2.01	1729
426	#318	17.20	0.49	1.21	1.87	285	2.41	1272
427	#320	7.86	0.33	2.25	4.85	273	1.92	950
428	#321	13.34	0.63	1.12	1.64	390	1.18	1164
429	#322	24.04	0.17	1.54	8.47	17	2.79	1445
430	#324	13.67	0.22	1.06	2.46	88	31.80	1175
431	#325	35.95	0.01	0.19	3.62	113	3.89	1791
432	#326	32.01	0.23	2.07	3.61	118	4.39	1678
433	#327	9.00	0.15	0.26	2.41	292	5.46	1007
434	#328	11.58	0.46	2.85	9.95	31	3.78	1101
435	#332	24.11	0.12	1.59	10.62	25	1.25	1446
436	#333	31.82	1.07	1.91	3.82	15	2.18	1674
437	#334	22.43	0.11	1.16	1.79	237	1.62	1407
438	#336	30.87	0.09	3.09	3.27	39	19.80	1653
439	#339	19.84	0.00	1.58	9.48	477	1.20	1341
440	#340	31.77	0.51	2.00	8.63	12	5.39	1674
441	#342	16.28	0.18	1.22	5.39	201	4.90	1248
442	#343	32.10	0.45	1.99	7.95	44	7.68	1683
443	#345	11.71	0.29	2.83	5.41	53	5.25	1111
444	#347	39.48	0.09	1.69	5.41	277	1.23	1911
445	#349	5.74	0.12	0.26	0.01	204	1.72	789
446	#350	22.20	0.06	0.54	1.70	582	6.82	1401
447	#351	9.90	0.07	1.77	6.48	162	3.15	1042
448	#352	24.36	0.33	1.44	6.75	94	6.91	1453
449	#355	13.21	0.23	3.08	0.92	347	2.29	1160
450	#357	25.52	0.26	0.75	10.74	159	4.25	1487
451	#358	34.12	0.28	2.10	2.63	22	3.92	1731
452	#361	24.46	0.19	1.27	3.17	48	51.15	1461
453	#363	10.01	0.02	1.75	5.40	146	3.81	1045

454	#364	24.38	0.10	1.68	2.33	31	2.58	1462
455	#366	6.28	0.06	0.25	3.46	145	2.12	848
456	#367	6.72	0.10	0.34	1.25	100	7.21	884
457	#370	29.80	0.22	0.47	9.38	139	2.51	1618
458	#371	58.74	0.38	0.05	1.71	123	2.04	2681
459	#372	39.28	0.14	1.10	13.63	80	4.59	1902
460	#375	7.00	0.07	1.70	2.46	262	3.61	904
461	#376	5.80	0.11	0.89	3.84	265	3.46	800
462	#380	20.19	0.08	0.47	6.13	1017	0.93	1352
463	#382	24.47	0.19	1.84	1.20	39	3.88	1464
464	#383	11.07	0.16	1.50	9.22	214	1.48	1084
465	#385	31.12	0.39	0.73	7.39	136	3.23	1658
466	#386	29.94	0.06	0.18	6.37	59	0.97	1625
467	#388	44.34	0.05	0.48	2.75	509	5.63	2086
468	#393	15.87	0.09	1.09	5.73	75	0.98	1238
469	#394	10.09	0.45	1.73	1.82	148	4.63	1047
470	#399	27.15	0.55	0.12	5.05	228	2.44	1533
471	#400	18.01	0.21	1.23	4.23	296	79.48	1294
472	#401	31.90	0.34	2.16	3.32	26	5.05	1681
473	#402	12.70	0.71	0.73	7.20	96	3.22	1146
474	#403	21.51	0.80	0.47	5.92	134	4.18	1385
475	#405	12.67	0.04	0.95	6.23	298	8.93	1144
476	#407	19.21	0.16	1.00	6.82	57	3.06	1325
477	#408	21.84	0.18	1.35	2.42	301	13.63	1393
478	#410	10.23	0.23	1.69	7.24	143	48.63	1054
479	#415	40.60	0.17	1.25	2.03	28	0.87	1952
480	#417	33.34	0.28	1.29	4.04	35	7.11	1714
481	#419	9.87	0.35	0.70	6.86	402	4.62	1040
482	#420	21.39	0.10	0.85	5.06	275	2.44	1381
483	#421	27.46	0.34	1.07	8.93	44	3.31	1536
484	#422	24.07	0.05	1.86	10.54	24	1.20	1446
485	#425	12.59	0.06	1.39	13.03	25	5.89	1141
486	#428	19.00	0.30	0.66	4.71	220	50.35	1320
487	#429	27.73	0.22	1.02	8.21	43	49.97	1555
488	#431	34.85	0.67	0.74	14.84	59	6.75	1754
489	#434	42.53	0.10	1.73	1.15	357	1.02	2021
490	#435	25.76	0.05	2.59	15.77	205	3.66	1495
491	#438	30.52	0.14	0.09	1.24	64	1.29	1649
492	#439	22.15	0.12	0.83	2.22	214	5.54	1401
493	#442	12.91	0.07	1.42	8.03	301	2.65	1152
494	#443	36.47	0.08	0.79	2.29	517	1.45	1811
495	#444	43.75	0.19	0.57	3.42	112	1.14	2062
496	#445	31.39	0.50	0.82	17.46	108	2.45	1664
497	#447	23.27	0.30	2.07	2.99	219	2.33	1427
498	#449	6.81	0.08	0.67	2.22	349	2.71	890
499	#452	30.25	0.15	0.09	5.47	51	3.87	1639
500	#454	24.69	0.46	1.49	2.96	100	13.93	1464
501	#458	39.90	0.29	1.28	1.29	228	1.44	1928
502	#460	37.07	0.05	1.44	2.33	37	7.39	1831
503	#461	31.55	0.15	0.80	3.30	176	14.84	1671
504	#464	58.75	0.14	0.14	1.24	112	2.52	2681
505	#465	14.90	0.24	1.85	6.21	821	2.06	1212
506	#466	6.93	0.05	1.53	1.00	213	2.75	900
507	#467	17.93	0.25	1.59	1.34	322	4.15	1292
508	#470	19.95	0.53	1.73	4.86	58	10.54	1345
509	#471	26.19	0.13	0.26	6.62	122	2.88	1506
510	#472	26.34	0.15	1.79	3.23	127	6.60	1510
511	#473	10.94	0.49	1.52	4.84	177	3.45	1079
512	#475	28.62	0.14	0.59	1.26	17	0.85	1578
513	#478	26.27	0.04	0.82	3.19	157	2.07	1508
514	#480	24.47	0.11	1.97	1.16	25	4.03	1464
515	#484	32.58	0.53	0.92	2.10	92	4.91	1694
516	#485	29.17	0.31	0.30	3.29	80	3.05	1596
517	#487	16.40	0.06	0.90	9.97	189	25.23	1251
518	#488	7.28	0.21	1.84	10.80	197	5.50	918
519	#490	10.87	0.29	1.53	5.35	169	57.43	1077
520	#491	24.13	0.16	2.02	3.10	16	3.26	1448
521	#492	13.41	0.12	2.99	2.18	115	2.40	1166
522	#495	10.75	0.22	1.56	3.17	179	4.19	1074
523	#496	43.60	0.07	0.11	0.35	53	3.65	2060
524	#497	31.49	0.37	1.06	3.46	20	1.79	1666
525	#499	9.07	0.21	0.74	2.67	131	27.22	1010
526	#501	12.54	0.19	0.67	3.61	177	1.16	1139
527	#507	29.15	0.17	0.92	4.99	65	3.19	1598
528	#508	9.13	0.14	2.89	2.93	93	2.28	1012
529	#511	29.01	0.14	1.57	14.65	70	15.77	1594

530	#513	24.04	0.02	2.06	1.02	10	9.07	1445
531	#515	43.60	0.06	0.18	7.43	54	1.24	2060
532	#516	13.47	0.26	2.96	6.79	107	5.63	1168
533	#517	26.59	0.21	0.82	1.27	162	48.32	1517
534	#520	27.54	0.12	1.29	4.79	55	3.92	1553
535	#521	6.93	0.20	0.48	7.68	66	1.96	900
536	#522	28.75	0.09	1.04	2.30	51	5.56	1585
537	#524	13.56	0.20	2.92	2.45	103	4.43	1173
538	#525	11.14	0.09	3.38	3.03	112	1.44	1082
539	#527	18.80	0.35	0.52	5.06	85	25.59	1315
540	#528	24.40	0.15	2.14	3.78	23	13.03	1461
541	#529	25.31	0.07	0.52	2.23	28	50.36	1480
542	#530	27.73	0.16	1.52	17.54	80	3.29	1551
543	#531	13.27	0.34	0.83	9.62	248	50.07	1162
544	#532	15.60	0.10	0.84	0.36	178	43.57	1232
545	#534	60.24	0.14	0.04	1.47	81	2.07	2740
546	#536	24.80	0.03	0.97	7.55	57	2.63	1466
547	#537	27.30	0.29	1.33	6.05	23	1.24	1536
548	#538	15.25	0.58	1.63	3.57	603	1.27	1222
549	#539	25.91	0.07	0.47	8.32	168	6.70	1499
550	#540	14.20	0.31	1.65	1.04	214	11.22	1191
551	#542	16.35	0.04	3.08	5.97	43	3.42	1251
552	#543	31.31	0.09	0.95	2.59	35	11.00	1663
553	#544	22.87	1.30	1.02	4.60	122	1.26	1417
554	#546	35.53	0.10	1.00	4.51	93	3.46	1780
555	#548	30.17	0.17	3.65	5.19	6	1.27	1632
556	#550	10.02	0.06	0.89	12.03	302	4.38	1045
557	#551	11.46	0.18	3.41	8.99	60	8.03	1099
558	#555	27.35	0.25	1.37	5.89	29	1.59	1540
559	#556	30.23	0.07	3.71	9.19	11	4.53	1632
560	#558	24.92	0.05	0.61	6.27	290	1.34	1470
561	#560	30.24	0.67	3.77	1.48	15	2.33	1632
562	#562	26.37	0.79	0.86	2.29	195	3.19	1512
563	#563	24.40	0.30	2.28	2.37	19	2.75	1462
564	#564	58.73	0.22	0.01	5.31	97	2.99	2681
565	#565	24.74	0.08	0.90	10.83	90	2.82	1464
566	#566	34.65	0.10	1.82	9.31	427	5.87	1747
567	#567	10.81	0.06	0.85	2.29	301	7.43	1074
568	#569	37.32	0.04	0.51	5.68	84	4.11	1846
569	#570	41.45	0.24	1.20	6.70	225	5.86	1980
570	#571	25.01	0.26	0.24	5.93	92	3.88	1472
571	#572	34.29	0.31	0.06	1.23	96	4.70	1745
572	#573	30.18	0.02	3.82	4.95	4	16.69	1634
573	#574	39.75	0.36	0.67	8.18	27	3.78	1921
574	#575	43.69	0.86	0.92	3.11	40	2.33	2062
575	#576	27.36	0.18	1.45	3.77	21	5.93	1538
576	#578	23.54	0.22	1.02	3.81	114	2.68	1433
577	#579	13.67	0.27	2.87	4.18	91	4.86	1175
578	#580	29.56	0.13	0.71	3.88	61	4.65	1603
579	#581	59.84	0.27	0.04	6.93	70	11.44	2725
580	#582	59.69	0.13	0.04	11.22	73	3.09	2719
581	#583	27.91	0.29	0.64	3.14	50	2.95	1555
582	#584	35.33	0.09	3.81	15.59	68	11.40	1774
583	#585	23.72	0.68	1.00	4.90	105	24.44	1437
584	#586	9.87	0.11	3.09	1.03	77	11.53	1040
585	#588	25.39	0.03	0.61	2.25	23	2.37	1481
586	#591	34.46	0.10	2.21	2.14	18	4.06	1747
587	#592	24.08	0.06	2.28	1.94	10	2.15	1448
588	#594	30.27	0.16	3.88	5.82	24	2.48	1632
589	#595	41.17	0.12	1.75	4.67	65	7.65	1971
590	#597	52.67	0.31	1.40	3.24	77	10.95	2445
591	#598	6.39	0.10	1.28	2.44	125	3.89	858
592	#599	31.02	0.08	1.06	19.80	138	11.00	1656
593	#600	41.94	0.25	0.24	3.45	485	2.28	2002
594	#601	7.99	0.02	2.48	2.38	221	7.43	957
595	#604	12.01	0.24	0.46	2.48	91	3.48	1119
596	#605	25.00	0.10	0.28	0.03	81	3.23	1470
597	#606	19.35	0.25	1.40	2.02	180	1.73	1329
598	#607	24.21	0.19	2.36	14.38	16	49.98	1448
599	#608	16.93	0.03	1.22	2.76	91	50.01	1265
600	#609	46.28	0.24	1.03	7.11	213	24.76	2154
601	#610	58.74	0.42	0.35	2.75	83	1.64	2681
602	#611	30.96	0.05	1.44	1.80	52	2.35	1658
603	#613	8.74	0.54	0.52	1.81	542	7.85	996
604	#614	30.94	0.10	1.54	3.52	54	3.91	1653
605	#617	30.25	0.37	3.95	1.16	17	4.28	1632

606	#618	16.53	0.20	1.64	2.88	246	3.17	1255
607	#619	43.68	0.21	1.02	4.17	44	2.35	2062
608	#620	40.18	0.10	0.67	9.98	111	7.55	1937
609	#621	20.80	0.29	1.14	4.39	245	1.23	1367
610	#622	59.67	0.33	0.13	7.12	65	6.05	2719
611	#623	10.73	0.23	0.63	5.25	438	50.03	1072
612	#624	30.33	0.12	0.04	13.18	29	2.84	1634
613	#625	27.65	0.31	1.62	6.25	31	1.79	1555
614	#626	27.26	0.14	1.51	14.10	20	1.71	1535
615	#627	30.83	0.10	1.48	9.75	37	15.56	1651
616	#628	50.67	0.08	0.93	3.11	253	4.96	2351
617	#629	29.61	0.28	2.24	3.74	97	6.12	1614
618	#630	60.25	0.13	0.13	2.54	78	3.27	2740
619	#632	23.81	0.19	3.09	6.75	73	1.86	1440
620	#633	52.67	0.36	1.44	2.18	73	29.55	2445
621	#634	27.46	0.56	1.56	7.59	19	1.03	1542
622	#635	58.74	0.21	0.26	8.76	81	0.99	2681
623	#640	28.88	0.34	1.10	3.34	42	6.26	1585
624	#641	13.81	0.20	2.81	1.05	81	3.73	1179
625	#644	4.55	0.10	0.79	3.49	129	2.22	589
626	#645	59.38	0.40	0.05	1.37	63	49.75	2706
627	#647	13.07	0.11	1.05	3.42	27	2.03	1154
628	#649	14.21	0.10	1.72	1.91	118	5.36	1191
629	#650	37.60	0.65	0.62	2.17	115	4.79	1848
630	#651	34.82	0.32	1.04	4.11	36	1.03	1754
631	#653	36.48	0.36	3.25	2.88	18	1.23	1811
632	#654	59.90	0.16	0.13	5.55	63	25.93	2725
633	#655	51.60	0.40	0.28	9.25	83	4.24	2395
634	#659	59.20	0.53	0.05	4.06	64	12.24	2696
635	#665	28.05	0.14	1.11	11.57	73	2.03	1558
636	#666	35.40	0.22	0.06	2.92	112	5.06	1776
637	#667	32.22	0.21	0.08	4.07	63	2.38	1684
638	#668	13.14	0.06	1.82	4.65	1399	3.62	1158
639	#669	27.29	0.40	1.60	3.39	17	4.51	1535
640	#670	13.87	0.18	2.78	4.80	77	49.38	1181
641	#673	8.66	0.40	2.76	3.36	67	21.15	993
642	#676	37.00	0.29	0.55	1.22	43	3.10	1828
643	#678	28.11	0.05	0.31	6.52	56	1.97	1562
644	#680	51.40	0.18	1.40	4.06	49	16.04	2385
645	#683	4.93	1.78	0.84	11.00	45	2.99	645
646	#685	13.94	0.28	2.75	1.34	74	1.70	1183
647	#686	28.29	0.21	1.11	3.80	70	2.38	1567
648	#687	16.39	0.09	2.89	4.03	147	3.24	1251
649	#691	33.80	0.61	0.76	2.00	121	1.87	1726
650	#692	41.13	0.59	0.44	5.09	80	3.14	1971
651	#694	24.12	0.13	2.59	3.96	11	24.06	1448
652	#696	25.27	0.14	0.78	16.46	160	2.42	1480
653	#697	14.01	0.23	2.72	1.74	76	6.27	1185
654	#698	28.88	0.34	1.48	6.38	9	2.36	1594
655	#700	43.68	0.54	1.24	4.00	31	12.03	2062
656	#701	43.68	0.09	1.19	2.06	29	5.68	2062
657	#702	43.68	0.11	1.15	6.70	29	1.86	2062
658	#703	28.84	0.63	1.55	2.04	22	10.81	1587
659	#705	37.03	0.06	0.62	15.20	150	5.97	1826
660	#707	5.93	0.32	0.51	10.23	219	13.18	822
661	#713	32.73	0.10	0.93	9.33	48	1.11	1697
662	#715	12.76	0.23	0.54	2.95	200	4.87	1146
663	#716	27.95	0.18	1.08	6.59	57	2.39	1558
664	#718	37.11	0.19	0.93	1.42	19	6.81	1833
665	#719	31.61	0.43	0.26	9.07	117	4.95	1671
666	#720	16.29	0.13	1.00	4.57	16	10.23	1248
667	#722	25.60	0.08	1.44	3.79	93	7.69	1489
668	#725	37.49	0.10	0.72	15.82	58	1.19	1842
669	#727	34.60	0.02	1.65	2.10	26	1.78	1747
670	#728	24.89	0.16	0.72	8.75	75	4.99	1466
671	#732	53.73	0.07	1.03	9.79	46	4.71	2486
672	#733	9.60	0.04	0.30	3.08	313	9.19	1030
673	#734	16.50	0.47	1.09	2.20	116	1.41	1255
674	#735	31.02	0.11	1.31	4.62	42	5.78	1656
675	#736	33.67	0.30	0.32	3.43	130	0.95	1722
676	#737	59.14	0.12	0.14	5.33	54	5.33	2694
677	#738	18.56	0.19	0.31	8.90	117	2.15	1308
678	#739	20.87	0.57	1.75	4.11	60	2.25	1370
679	#741	32.82	0.38	1.03	3.45	45	6.69	1701
680	#743	27.38	0.01	1.80	3.91	17	2.09	1535
681	#745	44.15	0.05	1.78	1.14	54	2.25	2084

682	#746	34.98	0.17	0.58	15.20	32	2.16	1760
683	#747	7.34	0.14	0.33	3.72	298	2.10	921
684	#748	34.78	0.99	1.10	3.46	26	15.49	1754
685	#749	4.69	0.33	0.56	5.34	31	3.88	622
686	#750	26.78	0.13	2.43	2.55	32	2.55	1523
687	#753	58.75	0.13	0.43	4.80	68	3.69	2681
688	#754	37.41	0.02	1.43	0.95	34	8.47	1842
689	#755	22.47	0.15	1.07	6.69	131	1.70	1407
690	#757	44.60	0.12	2.24	2.46	19	2.67	2096
691	#758	58.33	0.19	0.05	1.05	52	2.30	2668
692	#759	35.18	0.17	0.06	4.32	95	7.67	1767
693	#760	59.27	0.13	0.14	4.19	53	1.94	2701
694	#761	11.35	0.59	3.16	3.34	102	16.52	1097
695	#763	27.51	0.09	1.49	11.73	14	2.01	1542
696	#765	20.73	0.45	0.47	5.69	414	3.96	1365
697	#766	11.06	0.10	1.10	15.09	142	11.57	1084
698	#767	29.09	0.17	1.02	2.22	31	2.25	1596
699	#768	20.53	0.59	1.13	2.62	70	20.72	1360
700	#770	43.69	0.01	1.41	2.11	25	3.36	2062
701	#771	58.73	0.31	0.46	4.43	64	3.74	2681
702	#772	7.34	0.11	3.39	10.81	58	2.20	921
703	#773	17.09	0.26	0.58	4.87	63	10.83	1269
704	#774	58.16	0.12	0.05	9.48	54	4.80	2660
705	#775	24.44	0.17	1.80	4.41	35	5.92	1461
706	#776	27.35	0.17	1.93	10.46	14	1.82	1540
707	#777	8.45	0.10	2.40	10.92	92	1.91	979
708	#778	45.26	0.12	0.30	4.58	216	4.04	2120
709	#780	37.98	0.22	0.81	2.84	34	1.91	1861
710	#781	43.81	0.09	0.64	2.94	25	3.28	2067
711	#782	22.86	0.08	1.17	4.05	97	7.69	1417
712	#783	27.35	0.11	1.86	3.19	17	6.58	1538
713	#784	38.35	0.11	0.19	2.79	1223	5.82	1888
714	#785	34.02	0.17	3.59	4.37	6	2.50	1732
715	#786	34.05	0.06	3.70	10.36	7	4.14	1731
716	#787	38.65	0.13	0.83	3.48	13	3.33	1888
717	#790	32.59	1.14	1.31	7.69	31	3.49	1696
718	#791	32.74	0.19	1.57	4.96	80	47.24	1699
719	#792	57.88	0.02	0.05	4.53	51	3.95	2650
720	#793	30.94	0.16	1.64	2.50	31	15.59	1654
721	#794	4.77	0.10	0.78	4.69	13	5.55	622
722	#795	58.75	0.16	0.80	11.30	66	68.29	2681
723	#797	25.79	0.06	3.79	7.81	55	0.94	1495
724	#798	34.13	0.22	3.65	3.61	7	4.04	1732
725	#799	44.10	0.03	0.45	1.81	68	8.76	2077
726	#800	25.28	0.71	1.07	4.64	28	3.72	1478
727	#803	48.09	0.13	0.29	3.99	184	1.54	2235
728	#804	20.72	0.31	1.72	9.02	85	1.68	1365
729	#805	41.29	0.24	0.66	7.65	84	2.54	1975
730	#807	27.61	0.19	1.54	2.28	32	46.86	1546
731	#809	45.00	0.07	0.92	2.38	100	4.07	2111
732	#810	58.75	0.08	0.76	14.70	62	4.53	2681
733	#811	35.00	0.07	0.06	13.37	93	1.92	1765
734	#812	37.80	0.19	0.16	1.32	28	4.46	1855
735	#813	44.97	0.07	1.22	10.71	71	11.73	2108
736	#814	7.73	0.30	1.93	3.73	105	7.42	943
737	#815	31.43	0.37	0.33	1.84	63	10.74	1666
738	#816	15.25	0.40	1.16	1.57	120	2.04	1222
739	#817	27.37	0.90	1.98	2.39	12	45.06	1538
740	#818	52.67	0.09	1.59	5.05	54	6.23	2445
741	#819	17.14	0.73	1.13	11.00	108	7.08	1270
742	#820	7.42	0.74	1.88	3.26	103	1.32	925
743	#821	15.62	0.07	2.00	2.03	56	8.90	1234
744	#822	58.51	0.12	0.14	3.19	43	1.52	2670
745	#823	34.64	0.20	1.83	4.28	85	2.65	1749
746	#824	58.74	0.10	0.53	4.75	61	46.21	2681
747	#825	43.68	0.19	1.49	8.05	22	3.91	2062
748	#826	43.88	0.28	0.43	3.18	62	3.61	2069
749	#827	58.74	0.40	0.64	1.37	58	2.11	2681
750	#828	27.37	0.22	2.05	6.49	13	1.11	1536
751	#830	28.14	0.06	0.37	2.09	36	8.15	1564
752	#831	58.73	0.09	0.56	10.33	63	1.66	2681
753	#834	37.01	0.48	0.43	2.85	51	7.01	1826
754	#836	34.12	0.12	3.78	1.93	7	0.83	1732
755	#837	35.25	0.33	1.03	1.85	36	14.65	1769
756	#838	28.84	0.06	1.62	2.99	9	10.92	1585
757	#839	42.40	0.45	0.57	2.92	16	3.52	2016

758	#840	58.73	0.13	0.71	7.01	58	7.47	2681
759	#841	9.68	0.06	0.35	2.75	42	1.76	1032
760	#843	14.86	0.18	0.51	4.51	490	3.19	1210
761	#845	34.10	0.19	3.87	7.86	6	8.75	1731
762	#846	37.99	0.48	2.73	1.33	10	4.39	1864
763	#847	28.15	0.14	0.81	4.64	69	6.70	1564
764	#848	13.59	0.36	0.83	3.23	82	11.12	1173
765	#849	28.07	0.12	0.39	9.12	61	6.45	1562
766	#850	37.78	0.00	2.76	4.09	16	32.62	1853
767	#851	12.76	0.12	0.61	2.32	137	2.12	1146
768	#852	39.67	0.24	3.77	3.33	38	10.62	1918
769	#853	31.22	0.27	0.78	10.70	40	1.47	1661
770	#854	28.84	0.10	1.70	2.66	7	50.60	1585
771	#855	27.41	0.30	2.11	4.24	12	34.32	1540
772	#856	16.34	0.07	0.52	10.09	38	3.90	1249
773	#857	28.20	0.14	1.70	6.91	30	3.33	1567
774	#859	28.91	0.08	1.75	2.93	13	2.25	1594
775	#860	43.69	0.10	1.56	9.75	21	4.84	2062
776	#861	52.67	0.17	1.78	1.15	41	10.71	2445
777	#862	41.88	0.25	0.34	1.06	121	9.02	1997
778	#865	24.67	0.12	1.38	9.67	22	6.75	1461
779	#867	31.14	0.06	1.60	2.24	38	5.41	1659
780	#869	47.30	0.14	0.96	2.50	15	21.95	2187
781	#870	38.75	0.18	0.47	7.47	102	16.85	1888
782	#871	9.87	0.12	2.06	2.24	94	2.84	1040
783	#873	16.90	0.09	1.31	7.42	70	1.89	1265
784	#875	37.75	0.76	1.68	3.92	65	9.22	1855
785	#877	36.31	0.63	0.51	3.59	11	1.81	1804
786	#878	26.80	0.32	1.74	7.11	55	6.27	1523
787	#879	44.01	0.45	0.51	1.82	45	3.92	2074
788	#880	27.41	0.13	2.18	4.26	12	1.86	1544
789	#881	44.99	0.09	1.01	13.80	44	9.40	2108
790	#882	60.23	0.20	0.31	2.22	47	3.56	2740
791	#883	19.53	0.37	1.52	1.44	55	22.86	1334
792	#884	38.03	0.25	0.16	2.68	23	41.16	1864
793	#885	31.23	0.02	1.46	1.76	46	2.92	1659
794	#887	26.64	0.11	1.10	4.32	50	1.40	1517
795	#891	14.59	0.28	1.62	3.89	41	3.80	1201
796	#894	40.87	0.10	0.34	1.91	59	9.12	1961
797	#895	23.58	0.33	0.81	2.53	46	1.05	1435
798	#896	29.33	0.11	0.59	2.35	25	10.36	1603
799	#897	7.58	0.10	1.89	5.92	90	1.22	932
800	#898	30.94	0.06	1.72	6.12	26	10.80	1654
801	#899	16.88	0.44	0.84	3.37	85	15.20	1263
802	#901	32.43	1.04	1.97	2.37	445	8.14	1691
803	#903	24.71	0.12	1.24	1.85	23	2.02	1462
804	#905	6.80	0.30	1.05	1.14	34	23.00	884
805	#906	14.06	0.09	0.67	1.92	180	2.13	1187
806	#907	41.26	0.37	0.77	2.21	55	13.37	1973
807	#908	27.88	0.15	1.51	8.90	33	4.23	1555
808	#909	37.40	0.11	1.60	4.70	17	15.20	1842
809	#913	31.10	0.09	1.04	6.29	28	4.41	1658
810	#914	53.80	0.10	1.16	1.09	49	1.57	2489
811	#915	33.77	0.89	0.07	3.15	85	2.49	1722
812	#916	24.81	0.16	1.08	2.58	23	3.99	1468
813	#918	25.41	0.22	0.21	13.06	55	35.34	1483
814	#919	32.85	0.15	0.77	5.54	48	10.33	1702
815	#920	34.07	0.23	0.02	8.14	4	15.09	1734
816	#922	34.10	0.32	3.95	3.28	6	7.86	1732
817	#926	51.37	0.32	1.19	3.95	318	9.48	2382
818	#927	38.46	0.11	0.44	0.72	50	5.40	1881
819	#929	36.05	0.13	0.99	2.33	39	2.85	1798
820	#930	27.56	0.11	2.24	3.10	14	1.09	1542
821	#932	24.19	0.13	3.26	5.91	8	9.81	1446
822	#933	27.84	0.07	1.33	6.52	50	2.66	1555
823	#934	33.50	0.09	0.07	2.36	85	14.10	1717
824	#935	25.25	0.43	0.95	3.88	46	1.54	1478
825	#936	35.80	0.13	0.75	2.12	33	4.37	1789
826	#937	30.94	0.21	1.77	5.10	24	2.14	1654
827	#938	43.83	0.17	1.14	2.82	12	2.79	2067
828	#939	31.54	0.45	0.30	2.39	37	3.61	1669
829	#940	33.66	0.08	0.50	5.20	27	1.74	1722
830	#942	18.38	0.10	0.37	2.13	94	26.42	1304
831	#943	33.59	0.07	1.93	2.10	128	1.33	1719
832	#945	32.65	0.30	0.07	2.44	65	3.08	1701
833	#946	50.73	0.18	0.53	1.36	85	4.17	2354

834	#947	41.40	0.15	0.53	2.15	209	39.25	1978
835	#948	27.39	0.16	3.12	11.99	15	1.78	1536
836	#949	56.94	0.19	0.41	3.89	40	1.34	2613
837	#950	27.61	0.21	2.29	4.47	13	10.09	1555
838	#951	34.84	0.03	1.84	1.15	338	3.30	1754
839	#953	33.13	0.78	0.07	3.83	76	1.58	1711
840	#955	42.40	0.08	0.87	6.79	39	9.33	2016
841	#956	22.39	0.02	1.32	1.06	32	1.51	1406
842	#958	52.67	0.26	1.88	1.98	36	4.57	2445
843	#959	31.96	0.28	3.90	2.40	6	10.46	1683
844	#960	36.21	0.13	0.54	9.81	10	1.95	1802
845	#962	27.56	0.18	2.36	1.96	13	10.70	1540
846	#963	33.03	0.15	0.49	6.27	29	2.10	1707
847	#964	31.94	0.16	3.96	2.08	5	3.03	1683
848	#967	45.36	0.05	0.34	1.73	68	1.40	2120
849	#968	59.73	0.93	0.32	2.44	35	6.49	2717
850	#969	33.71	0.38	0.97	2.84	53	1.20	1724
851	#972	32.96	0.14	0.61	15.95	27	6.79	1704
852	#973	28.90	0.35	1.85	2.28	6	1.91	1594
853	#974	39.89	0.07	1.36	6.85	34	4.32	1923
854	#975	45.92	0.25	0.48	4.70	41	1.76	2142
855	#978	28.22	0.20	0.90	2.31	52	4.26	1567
856	#979	38.19	0.15	0.70	1.44	58	16.46	1866
857	#980	29.79	0.19	0.33	4.95	13	1.47	1621
858	#981	36.09	0.16	0.62	3.92	10	1.78	1798
859	#982	28.95	0.35	0.10	3.14	37	1.98	1598
860	#983	32.98	0.26	1.59	5.23	135	1.37	1706
861	#984	44.22	0.11	0.55	2.42	99	4.95	2082
862	#985	27.38	0.49	2.41	2.65	12	1.88	1538
863	#987	31.96	0.47	0.03	4.28	7	3.83	1683
864	#990	29.34	1.52	0.10	3.12	37	3.18	1605
865	#992	56.92	0.26	1.13	1.21	144	4.67	2606
866	#994	5.92	0.17	1.01	3.82	142	6.13	811
867	#995	34.65	0.11	1.56	5.62	17	14.22	1749
868	#997	5.94	0.14	0.70	7.44	75	15.82	822
869	#1000	59.57	0.27	0.32	10.09	31	1.42	2714
870	#1001	25.63	0.16	0.52	2.86	32	1.21	1489
871	#1002	13.54	0.15	0.54	4.14	94	6.85	1170
872	#1003	35.74	0.15	0.60	3.00	96	1.72	1789
873	#1005	4.80	0.43	0.17	1.95	799	2.46	622
874	#1008	40.62	0.19	0.23	2.61	106	1.21	1947
875	#1009	24.63	0.32	1.58	0.85	19	1.52	1462
876	#1011	27.55	0.41	2.47	2.15	11	5.05	1538
877	#1012	37.78	0.16	0.52	2.50	91	3.82	1853
878	#1013	52.67	0.19	1.97	2.25	32	1.37	2445
879	#1016	35.89	0.30	0.74	2.63	20	2.02	1791
880	#1018	40.24	0.35	1.00	1.86	18	1.45	1937
881	#1019	28.92	0.10	1.92	2.02	6	2.12	1596
882	#1020	4.54	0.06	0.58	6.47	137	3.45	589
883	#1021	56.96	0.13	1.01	7.14	191	1.06	2613
884	#1022	39.39	0.12	0.15	6.81	17	3.65	1907
885	#1025	31.56	0.27	0.08	3.86	53	6.25	1673
886	#1026	12.76	0.22	1.49	1.41	29	3.81	1146
887	#1027	27.35	0.26	2.57	1.92	6	4.02	1538
888	#1028	20.69	0.17	0.89	1.88	45	4.05	1363
889	#1030	35.89	0.62	0.40	2.06	29	8.51	1791
890	#1032	18.45	0.37	3.76	2.63	49	4.75	1306
891	#1034	59.45	0.16	0.33	14.94	34	9.75	2709
892	#1035	15.87	0.27	3.04	3.78	46	1.43	1240
893	#1036	38.19	0.20	0.47	1.79	72	4.70	1870
894	#1037	53.17	0.28	0.22	3.69	42	1.82	2466
895	#1038	38.46	0.29	0.59	0.98	23	1.32	1881
896	#1039	8.01	0.16	2.28	1.32	54	2.70	957
897	#1040	39.19	0.49	0.15	3.24	17	1.15	1899
898	#1041	30.93	0.20	1.86	5.55	21	14.38	1654
899	#1042	60.24	0.39	0.25	7.46	32	7.44	2738
900	#1044	35.36	1.93	0.86	2.52	89	3.24	1769
901	#1045	35.84	0.31	0.30	3.06	39	4.47	1789
902	#1046	36.13	0.15	0.75	3.65	36	1.24	1800
903	#1048	10.80	0.34	3.31	1.79	31	9.79	1074
904	#1049	32.48	0.17	2.02	6.62	415	9.67	1691
905	#1050	26.61	0.14	1.43	2.35	32	25.61	1517
906	#1051	29.61	0.51	0.10	1.54	38	30.06	1614
907	#1052	59.30	0.10	0.33	11.12	30	1.06	2704
908	#1053	36.73	0.26	0.27	6.26	37	5.91	1822
909	#1054	25.92	0.14	0.62	5.16	29	1.74	1499

910	#1056	58.43	0.61	0.36	1.52	36	5.62	2670
911	#1057	45.66	0.86	0.80	5.36	9	5.62	2130
912	#1058	6.20	0.54	1.34	1.66	30	8.90	843
913	#1059	19.47	0.47	3.69	1.86	23	14.70	1333
914	#1060	35.22	1.31	0.43	2.48	20	1.54	1769
915	#1061	23.21	0.16	1.38	2.25	524	2.22	1425
916	#1064	44.64	0.19	0.36	5.68	51	6.59	2096
917	#1065	27.53	0.38	2.65	1.47	9	1.85	1538
918	#1066	41.96	0.13	0.77	4.46	34	2.33	1999
919	#1067	39.25	0.10	1.66	2.17	55	11.30	1904
920	#1068	53.80	0.44	1.24	2.25	34	4.05	2489
921	#1069	52.16	0.32	1.04	1.70	100	2.06	2417
922	#1071	28.86	0.14	2.01	5.62	6	5.55	1594
923	#1073	52.46	0.41	0.13	2.01	18	5.29	2432
924	#1074	10.24	0.12	3.22	1.68	29	2.92	1054
925	#1076	39.74	0.42	1.05	2.06	41	3.17	1921
926	#1077	29.76	0.20	0.90	13.32	52	2.46	1618
927	#1079	30.99	0.60	0.08	1.89	47	7.08	1659
928	#1081	28.90	0.29	1.97	2.83	5	6.52	1594
929	#1082	10.01	0.19	2.88	7.67	45	1.33	1045
930	#1083	59.11	0.30	0.34	0.97	28	2.17	2696
931	#1084	26.57	0.38	1.51	1.51	77	2.66	1517
932	#1085	27.42	0.22	2.76	7.08	8	3.79	1544
933	#1088	46.29	0.30	0.39	1.59	42	4.69	2157
934	#1089	27.45	0.11	3.07	1.20	11	2.10	1536
935	#1090	28.09	0.21	0.73	1.45	42	4.64	1564
936	#1092	38.92	0.17	0.53	6.45	36	5.23	1890
937	#1093	34.66	0.14	2.05	1.78	17	3.86	1747
938	#1095	39.63	0.19	0.38	3.97	28	2.31	1911
939	#1096	50.82	0.39	1.83	2.58	21	11.99	2357
940	#1097	53.79	0.16	1.29	1.62	65	3.24	2489
941	#1098	27.52	0.16	2.83	5.29	8	3.59	1551
942	#1099	38.24	0.19	0.57	13.61	30	3.39	1868
943	#1100	36.36	0.11	3.27	8.15	10	1.09	1809
944	#1102	59.92	0.26	0.62	8.51	20	1.31	2727
945	#1103	43.80	0.13	1.78	5.29	48	3.97	2067
946	#1106	31.56	0.21	0.97	5.40	26	7.81	1669
947	#1107	52.67	0.61	2.18	1.54	26	3.82	2445
948	#1108	28.03	0.23	0.80	2.33	27	5.69	1560
949	#1109	44.97	0.20	0.36	2.39	44	28.63	2108
950	#1110	27.87	0.24	3.04	2.37	64	5.20	1555
951	#1111	28.94	0.58	2.09	1.40	5	1.85	1596
952	#1114	30.96	0.55	1.97	1.68	20	3.11	1654
953	#1115	8.87	0.45	2.83	1.54	30	3.14	1002
954	#1116	41.63	0.47	0.78	3.87	18	1.15	1990
955	#1117	27.64	0.40	2.07	1.21	16	2.58	1551
956	#1118	9.86	0.59	1.25	4.33	20	2.83	1040
957	#1119	35.29	0.52	1.69	2.76	30	5.10	1769
958	#1120	17.67	0.45	0.70	0.98	54	0.92	1285
959	#1121	41.41	0.25	0.76	4.91	91	1.44	1980
960	#1122	37.34	0.40	0.38	3.12	126	6.29	1837
961	#1125	44.82	0.27	0.36	2.05	41	8.95	2103
962	#1128	21.31	0.40	2.38	1.56	42	7.14	1380
963	#1129	27.47	0.19	2.89	4.47	7	6.47	1536
964	#1131	35.88	0.22	0.61	0.74	19	13.17	1791
965	#1132	28.86	0.23	2.14	2.38	5	13.32	1596
966	#1133	28.90	0.43	2.31	3.69	202	8.99	1582
967	#1134	19.82	1.72	3.69	1.14	18	13.06	1341
968	#1137	28.90	0.38	2.26	4.22	164	8.05	1591
969	#1138	60.16	0.38	0.69	2.94	31	13.80	2735
970	#1140	5.96	0.13	0.89	0.86	98	4.09	822
971	#1141	27.23	0.42	3.20	3.45	49	15.95	1535
972	#1142	24.73	0.43	1.74	4.78	17	22.48	1464
973	#1143	27.59	0.39	2.72	1.97	8	4.28	1551
974	#1144	29.21	0.32	1.78	5.42	22	5.29	1605
975	#1148	20.14	0.43	1.14	6.04	40	5.68	1350
976	#1150	47.53	0.48	3.56	7.92	20	21.36	2210
977	#1151	32.76	0.44	1.19	8.10	14	23.12	1699
978	#1152	22.18	0.32	0.65	9.14	51	4.51	1401
979	#1153	34.93	0.13	1.64	5.89	470	2.37	1756
980	#1154	48.95	0.18	1.21	6.74	180	1.05	2281
981	#1157	48.12	0.32	1.07	2.61	143	7.46	2230
982	#1158	36.56	0.29	0.56	3.08	10	13.61	1817
983	#1160	60.15	0.31	0.72	1.43	30	14.94	2732
984	#1161	58.16	0.20	0.37	4.36	28	6.62	2657
985	#1162	27.48	0.18	2.97	0.95	7	6.37	1536

986	#1167	59.95	0.15	0.70	2.75	24	5.16	2727
987	#1168	31.09	0.13	2.07	3.97	27	22.52	1656
988	#1169	9.26	0.11	2.66	4.13	32	12.69	1017
989	#1170	34.93	0.08	1.54	1.81	697	2.32	1763
990	#1171	57.60	0.06	3.29	2.24	20	4.36	2637
991	#1172	45.53	0.04	0.34	3.50	53	0.95	2128
992	#1173	48.70	0.01	0.50	0.69	17	2.75	2258
993	#1176	22.03	0.29	1.49	4.67	54	3.97	1396
994	#1177	33.07	0.36	1.05	2.92	20	4.13	1706
995	#1178	24.17	0.40	3.92	5.26	6	1.81	1450
996	#1181	59.56	0.33	0.39	7.18	41	2.24	2714
997	#1183	57.73	0.30	0.37	1.36	27	3.51	2644
998	#1184	40.37	0.42	0.14	3.22	17	0.69	1947
999	#1186	15.47	0.27	0.17	2.49	14	4.66	1230
1000	#1187	17.36	0.38	3.32	4.04	28	2.92	1278
1001	#1188	59.50	0.22	0.74	0.81	22	5.16	2709
1002	#1190	29.09	0.35	2.28	3.59	44	7.10	1596
1003	#1191	40.84	0.17	0.81	2.11	17	1.36	1961
1004	#1192	42.65	0.44	0.76	4.93	12	3.22	2024
1005	#1193	57.49	0.32	0.39	0.12	24	2.49	2634
1006	#1194	25.61	0.29	0.21	0.60	32	4.05	1489
1007	#1195	27.64	0.41	2.59	0.26	8	0.82	1551
1008	#1196	29.28	0.36	1.59	3.64	37	3.59	1603
1009	#1198	42.59	0.24	0.57	1.21	42	2.11	2024
1010	#1199	28.94	0.43	2.93	4.38	29	4.93	1594
1011	#1200	26.64	0.31	1.99	2.74	23	1.77	1517
1012	#1201	53.84	0.37	0.31	0.92	33	3.42	2489
1013	#1202	25.93	0.19	0.88	4.96	34	0.53	1499
1014	#1203	24.73	0.40	1.82	3.19	12	2.65	1464
1015	#1204	38.86	0.28	0.75	2.06	15	4.78	1888
1016	#1205	40.60	0.35	0.80	1.57	9	1.24	1949
1017	#1206	39.47	0.22	1.88	4.47	47	3.06	1909
1018	#1207	56.30	0.34	1.22	0.73	93	4.50	2582
1019	#1208	24.82	0.43	2.30	2.37	100	2.30	1470
1020	#1209	33.19	0.30	0.54	3.71	11	1.21	1709
1021	#1210	47.69	0.38	0.54	1.14	22	4.32	2218
1022	#1211	26.84	0.26	3.26	4.20	109	2.75	1525
1023	#1212	29.07	0.37	1.77	2.94	12	0.93	1596
1024	#1213	51.40	0.28	1.50	0.87	28	4.88	2385
1025	#1214	44.27	0.41	0.94	3.49	41	3.09	2084
1026	#1215	44.33	0.20	0.79	4.81	33	2.04	2086
1027	#1216	22.15	0.23	3.18	1.96	4	1.56	1399
1028	#1217	58.31	0.25	0.83	5.22	29	4.40	2668
1029	#1218	38.48	0.43	0.70	6.05	18	0.72	1883
1030	#1219	25.14	0.34	1.18	7.64	16	2.38	1474
1031	#1220	25.34	0.37	1.55	8.31	19	3.67	1481
1032	#1221	58.34	0.39	0.28	9.07	21	1.15	2668
1033	#1222	24.98	0.15	1.42	5.69	15	4.21	1468
1034	#1223	7.40	0.42	1.16	6.73	27	2.93	925
1035	#1224	7.38	0.43	0.93	2.61	46	0.87	921
1036	#1226	27.43	0.38	3.49	3.08	5	3.51	1540
1037	#1227	28.92	0.31	2.51	1.43	5	4.80	1596
