# Supplementary material for: Advancing fecal volatilome profiling by comprehensive two-dimensional gas chromatography-time-of-flight mass spectrometry and image pattern recognition
Source: Anal Bioanal Chem. 2026 Jan 8;418(10):3037–58. doi: 10.1007/s00216-025-06280-6 (PMC13144212; doi:10.1007/s00216-025-06280-6)
Supplement: Supplementary file 2 — Supplementary Material 2 (PDF 434 KB) [file 216_2025_6280_MOESM2_ESM.pdf]

Electronic Supplementary Material

Advancing Fecal Volatilome Profiling by Comprehensive Two-Dimensional Gas Chromatography-Time of Flight Mass Spectrometry and Image Pattern Recognition

Fulvia Trapani[1], Andrea Caratti[1], Erica Liberto[1], Luca Cocolin[2], Ilaria Goitre[3], Valentina Ponzo[3], Simona Bo[3], Chiara Cordero[1*], Ilario Ferrocino[2]

Authors' affiliation:

[1]Dipartimento di Scienza e Tecnologia del Farmaco, Università di Torino, Via Giuria 9, 10125 Turin, Italy

[2]Dipartimento di Scienze Agrarie, Forestali e Alimentari, Università di Torino, Largo P. Braccini 2, 10095 Grugliasco (TO), Italy

[3]Dipartimento di Scienze Mediche, Università di Torino, Corso Dogliotti 14, 10125 Turin, Italy

*Address for correspondence:

Prof. Chiara Cordero - Dipartimento di Scienza e Tecnologia del Farmaco, Università degli Studi di Torino, Via Pietro Giuria 9, I-10125 Torino, Italy – e-mail: chiara.cordero@unito.it; phone: +39 011 6702197

Supplementary Figure 1 – SF1: Principal Component Analysis (PCA) scores plot based on VOC fingerprinting of fecal samples, showing distribution of samples across experimental groups (T1VP1, T2VP1, T1VP0, T2VP0). Colored ellipses represent the 95% confidence interval for each group. Quality control (QC) samples, highlighted separately, cluster tightly and confirm analytical reproducibility.

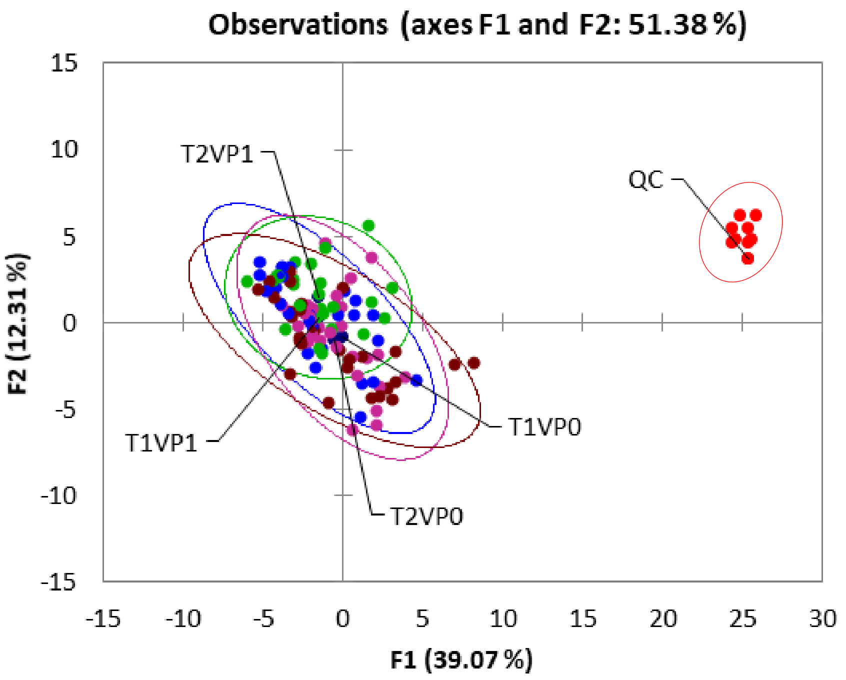

Supplementary Figure 2 – SF2: Number of detected fecal headspace VOC features (SNR > 50) obtained with seven HS-SPME fibers. Bars report total feature counts (mean of triplicates) after exclusion of interferents (coating residues, column bleed).

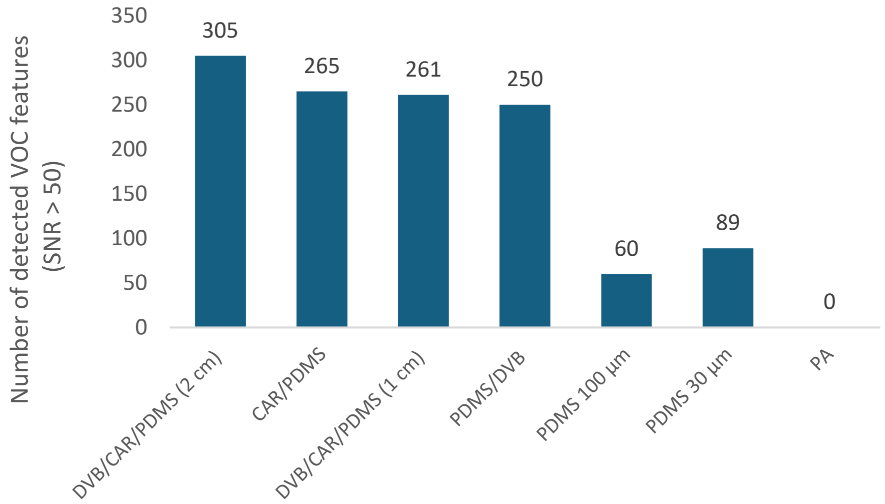

Supplementary Figure 3 – SF3: PCA scores plot based on samples collected two weeks after gluten reintroduction (T2); the VOCs matrix comprises 25 Fisher-filtered features.

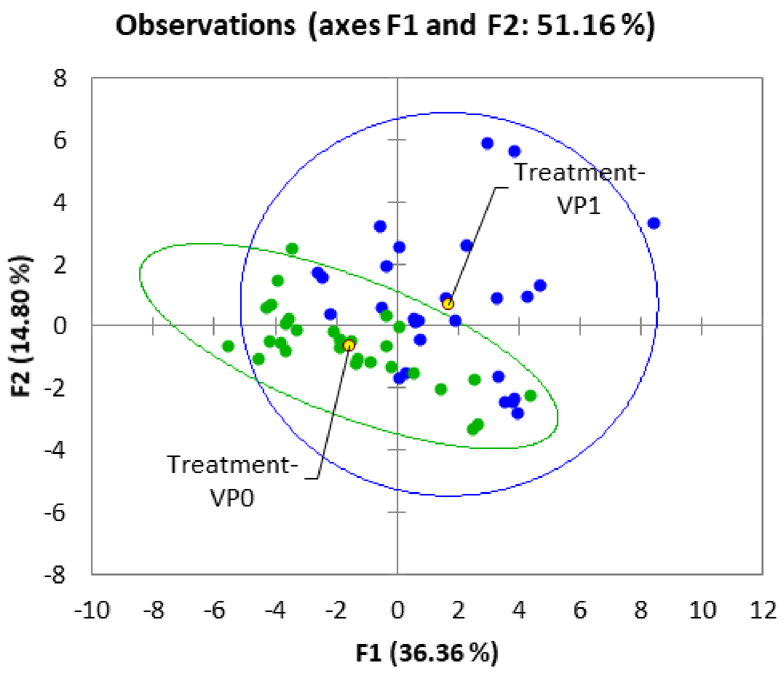

Supplementary Figure 4 – SF4: Box plots of relative signal intensities (GC×GC–TOF MS) for indole, octanal and γ-caprolactone at T1 (four weeks of gluten withdrawal). Probiotic (VP1, blue) versus placebo (VP0, orange). Each box shows the interquartile range with median; whiskers extend to 1.5×IQR. Indole and octanal medians are lower in VP1, indicating reduced proteolytic fermentation and lipid-oxidation byproducts under probiotic treatment. γ-Caprolactone shows a modest decrease in VP1, reflecting early shifts in microbial lipid metabolism.

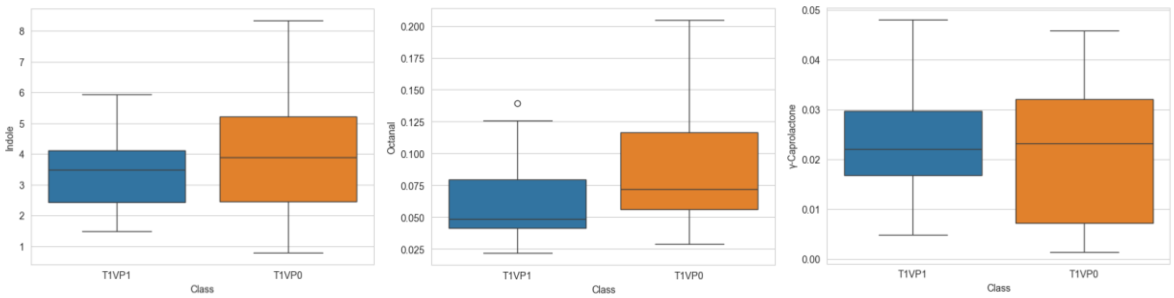

Supplementary Table 1 - ST1: Sample coding and metadata used in the HS-SPME/GC×GC-TOF MS batches. Columns report the datafile name (Datafile), anonymized sample identifier (Sample code), time point (Time point), treatment arm (Treatment), combined factor (Combined)and plain-treatment arm (Treatment label). T1 corresponds to the gluten-free diet phase (4 weeks up to sampling); T2 corresponds to the free-diet phase (2 weeks between T1 and T2). VP0 = Placebo; VP1 = Probiotics.

Sample ID	Sample code	Time point	Treatment	Combined	Treatment label
1A_T1_VP1	1A	T1	VP1	T1VP1	Probiotics
1B_T2_VP1	1B	T2	VP1	T2VP1	Probiotics
2A_T1_VP1	2A	T1	VP1	T1VP1	Probiotics
2B_T2_VP1	2B	T2	VP1	T2VP1	Probiotics
3A_T1_VP1	3A	T1	VP1	T1VP1	Probiotics
3B_T2_VP1	3B	T2	VP1	T2VP1	Probiotics
4A_T1_VP1	4A	T1	VP1	T1VP1	Probiotics
4B_T2_VP1	4B	T2	VP1	T2VP1	Probiotics
5A_T1_VP1	5A	T1	VP1	T1VP1	Probiotics
5B_T2_VP1	5B	T2	VP1	T2VP1	Probiotics
6A_T1_VP1	6A	T1	VP1	T1VP1	Probiotics
6B_T2_VP1	6B	T2	VP1	T2VP1	Probiotics
A17_T1_VP1	A17	T1	VP1	T1VP1	Probiotics
A18_T2_VP1	A18	T2	VP1	T2VP1	Probiotics
A23_T1_VP1	A23	T1	VP1	T1VP1	Probiotics
A24_T2_VP1	A24	T2	VP1	T2VP1	Probiotics
A27_T1_VP1	A27	T1	VP1	T1VP1	Probiotics
A28_T2_VP1	A28	T2	VP1	T2VP1	Probiotics
A32_T1_VP1	A32	T1	VP1	T1VP1	Probiotics
A33_T2_VP1	A33	T2	VP1	T2VP1	Probiotics
A39_T1_VP1	A39	T1	VP1	T1VP1	Probiotics
A40_T2_VP1	A40	T2	VP1	T2VP1	Probiotics
A56_T1_VP1	A56	T1	VP1	T1VP1	Probiotics
A57_T2_VP1	A57	T2	VP1	T2VP1	Probiotics
K4_T1_VP1	K4	T1	VP1	T1VP1	Probiotics
K5_T2_VP1	K5	T2	VP1	T2VP1	Probiotics
A10_T1_VP0	A10	T1	VP0	T1VP0	Placebo
A11_T2_VP1	A11	T2	VP1	T2VP1	Placebo
A13_T1_VP0	A13	T1	VP0	T1VP0	Placebo
A14_T2_VP0	A14	T2	VP0	T2VP0	Placebo
A20_T1_VP0	A20	T1	VP0	T1VP0	Placebo
A21_T2_VP0	A21	T2	VP0	T2VP0	Placebo
A43_T1_VP0	A43	T1	VP0	T1VP0	Placebo
A44_T2_VP0	A44	T2	VP0	T2VP0	Placebo
A7_T1_VP0	A7	T1	VP0	T1VP0	Placebo
A8_T1_VP0	A8	T1	VP0	T1VP0	Placebo
A80_T1_VP0	A80	T1	VP0	T1VP0	Placebo
A81_T2_VP0	A81	T2	VP0	T2VP0	Placebo
A82_T1_VP0	A82	T1	VP0	T1VP0	Placebo
A83_T1_VP0	A83	T1	VP0	T1VP0	Placebo
A84_T2_VP0	A84	T2	VP0	T2VP0	Placebo
A85_T2_VP0	A85	T2	VP0	T2VP0	Placebo
A86_T2_VP0	A86	T2	VP0	T2VP0	Placebo
A87_T2_VP0	A87	T2	VP0	T2VP0	Placebo
A88_T1_VP0	A88	T1	VP0	T1VP0	Placebo
A89_T1_VP0	A89	T1	VP0	T1VP0	Placebo
A90_T1_VP0	A90	T1	VP0	T1VP0	Placebo
A91_T1_VP0	A91	T1	VP0	T1VP0	Placebo
A92_T2_VP0	A92	T2	VP0	T2VP0	Placebo
A94_T2_VP0	A94	T2	VP0	T2VP0	Placebo
B7_T1_VP0	B7	T2	VP0	T2VP0	Placebo
B8_T2_VP0	B8	T2	VP0	T2VP0	Placebo
K7_T1_VP0	K7	T1	VP0	T1VP0	Placebo
K8_T2_VP0	K8	T2	VP0	T2VP0	Placebo

Supplementary Table 2 – ST2: provided as *pdf file

list of targeted compounds annotated based on I^T and spectral similarity together with untargeted features (#n) indicated with with retention times (^1t_R and ^2t_R), relative standard deviation (RSD%), I^T experimentally estimated and for targeted analytes tabulated I^T in NIST spectral library.
